# The rhythm of mental health: the relationship of chronotype with psychiatric trait dimensions and diurnal variation in psychiatric symptoms

**DOI:** 10.1038/s41398-024-02943-7

**Published:** 2024-06-04

**Authors:** Leonie J. T. Balter, Benjamin C. Holding, Predrag Petrovic, John Axelsson

**Affiliations:** 1 Department of Clinical Neuroscience, Karolinska Institutet, Stockholm, 171 65 Sweden; 2 Department of Psychology, Stress Research Institute, Stockholm University, Stockholm, 114 19 Sweden; 3 Department of Sociology, University of Copenhagen, Copenhagen, DK 1014 Denmark

**Keywords:** Human behaviour, Neuroscience, Psychiatric disorders

## Abstract

To advance the emergence of circadian-based therapies, this study characterized how psychiatric symptoms fluctuate across the day and vary between individuals. Using a dimensional approach, we determined how chronotype relates to 13 psychiatric traits, and modeled the temporal development of symptoms throughout the day using generalized additive mixed effects models. In this preregistered study, a subclinical sample completed 13 psychiatric trait scales and a chronotype scale at baseline (*N* = 515, *n* = 404 women, 109 men, *n* = 2 non-binary, *M* age = 32.4 years, range 18–77), followed by 22 psychiatric symptoms and behaviors rated repeatedly between ~08:00-00:00 (*n* = 410). Key findings are that 11 out of 13 psychiatric traits were associated with being an evening-type, ranging from depression to obsessive comulsive disorder, social anxiety, and delusional ideation, while only mania was associated with being a morning-type. Four distinct psychiatric trait factors were identified, each predicting worse symptom levels throughout the day. *Fatigue-related symptoms* exhibited strong time-of-day changes with evening-types experiencing worse fatigue in the morning and morning-types in the evening. Evening-types had considerably lower *drive and motivation* than morning-types from morning to early evening. Evening-types also had more pronounced *negative emotional symptoms* and *ADHD-type symptoms* in the evening, particularly among those high in psychiatric trait factors. These findings identified important research targets that hold promise for improving mental health outcomes, such as strategies to boost morning motivation. Furthermore, the results emphasize the relevance of incorporating circadian factors, including chronotype, into translational psychiatric research and interventions.

## Introduction

Nearly 1 billion people worldwide suffer from a mental disorder, leading to reduced productivity, reduced quality of life, and tremendous health care costs [1]. Emerging therapies that utilize time-of-day to strengthen treatment effects in psychiatry give promising results [2], but more effective and widespread therapeutic applications in mental health care are dependent on a better understanding of how and why psychiatric symptoms vary across the day.

Life on earth has evolved in synchrony with the day-night cycle. Most species have a circadian system that modulates a multitude of biological processes across the 24-h day [3, 4]. This involves regulation of the sleep/wake cycle, local expression of clock genes, hormone levels, synaptic functioning, and activity in brain regions regulating mood and arousal [5–9]. Circadian disruptions, common in shift work, increase risk for developing psychiatric disorders [5, 10, 11]. There is also compelling evidence of disrupted and shifted circadian rhythms in mood disorders [5, 12–14] and it is increasingly acknowledged in other psychiatric disorders such as schizophrenia [15] and attention deficit hyperactivity disorder (ADHD) [16]. Notably, symptoms relating to mood disorders are not static but can fluctuate across the 24 h period, with patterns varying from worse symptoms in the morning, afternoon, or evening [17–19]. Individual differences in the temporal expression of circadian-regulated output, referred to as an individual’s chronotype [20], can influence psychiatric symptom patterns [21]. Chronotype, reflecting whether a person is a morning-type (“morning lark”), intermediate-type, or evening-type (“night owl”), manifests in individuals functioning and feeling better at different times of day [22]. We are, however, only beginning to grasp the extent to which chronotype influences diurnal (i.e., time-of-day) patterns of psychiatric symptoms and behaviors.

Besides well-established genetic and physiological differences between chronotypes [23], there are also phenotypic health differences, e.g., evening-types are more likely to suffer from mood problems and psychiatric problems beyond mood [24, 25]. However, due to contradictory findings and/or limited scientific attention (e.g., for anxiety disorders, eating disorders, psychosis, ADHD, autism spectrum disorders, mania) these latter relationships remain less clear (reviewed in refs. [24, 26]).

The aim of the current preregistered study was therefore threefold: 1) to determine the relationships between chronotype and common psychiatric traits; 2) to characterize how psychiatric symptoms and behaviors develop across the day; 3) to determine how chronotype and transdiagnostic features of psychiatric disturbances predict these diurnal patterns. Using the Research Domain Criteria (RDoC) [27] we conceptualize psychiatric disturbances on a continuum in order to describe the full range of psychiatric variation, from normal to sub-clinical and clinical levels. We expected diurnal variation in psychiatric symptoms and behaviors to depend on chronotype, manifested by worse levels at suboptimal times of arousal, i.e., worse morning symptoms in evening-types and worse evening symptoms in morning-types, in line with the proposed “synchrony effect” [28]. We also expected that individuals with poorer mental health traits would exhibit more severe psychiatric symptoms across the day.

## Materials and methods

### Participants

Data for the factor analysis of the psychiatric symptoms and behaviors was available for 605 adult participants. In total, 515 participants (404 women, 109 men, 2 non-binary; *M* age = 32.4, *SD* = 9.5, range 18–77) completed the entire baseline session and passed the attention checks (as per preregistration, *n* = 6 were excluded because of possible inattention) (link to preregistration: 10.17605/OSF.IO/8TM5U). The next day, 410 participants completed two or more of the timepoints (n = 27 completed only one timepoint and were excluded), of which 73 morning-types, 243 intermediate-types, and 94 evening-types. Participants were recruited via an online recruitment platform for academic research (Prolific.co). Individuals residing in the United Kingdom, fluent in English, 18 years or older, and an approval rate of ≥99% in previous participations were invited to participate. Compensation was £7.50 per hour with a bonus of £5 if all timepoints were completed. See Table 1 and Supplement for further information.

**Table 1:** Self-reported psychiatric diagnoses and medication intake, presented as counts (%*)* and percentages (%).

*N*	515
Self-reported psychiatric diagnosis	
Mood disorder, *n* (%)	16 (3.1%)
Anxiety disorder, *n* (%)	15 (2.9%)
Schizoaffective disorder, *n* (%)	1 (0.2%)
Compulsive disorder, *n* (%)	3 (0.6%)
Personality disorder, *n* (%)	5 (1.0%)
Post-Traumatic Stress Disorder, *n* (%)	2 (0.4%)
Other medical diagnosis, *n* (%)	58 (11.3%)
Medication	
Anti-depressant/anxiety, *n* (%)	69 (13.4%)
Anti-psychotic, *n* (%)	3 (0.6%)
Mood stabilizer, *n* (%)	1 (0.2%)
Sleep, *n* (%)	2 (0.4%)
Other medication (non-psychiatric), *n* (%)	131 (25.4%)

Individuals may present with multiple diagnoses and take multiple medications. Mood disorder includes depressive disorder and bipolar disorder. Anxiety disorder includes generalized anxiety disorder and panic disorder. Compulsive disorder includes obsessive-compulsive disorder (OCD) and eating disorder. Personality disorder includes borderline personality disorder. No cases of attention-deficit/hyperactivity disorder (ADHD), autism spectrum disorders, or psychosis were reported. Other medical diagnosis includes e.g., asthma, hypertension, diabetes, Crohn’s disease, rheumatoid arthritis, inflammatory bowel disease, arrhythmia, psoriasis, fibromyalgia, sickle cell disease, osteoporosis, chronic pain, fibromyalgia, under- or over-active thyroid. Anti-depressant/anxiety medication include selective serotonin reuptake inhibitors (SSRIs), serotonin-norepinephrine reuptake inhibitors (SNRIs), tricyclic antidepressants (TCAs), and other. Other medication covers a range of non-psychiatric medications such as antihypertensives, statins, diuretics, antihistamines, proton pump inhibitors, asthma medications, immunosuppressants. Contraceptives are omitted.

### Study design

The study was carried out across two weekdays with seven measurement timepoints, consisting of a baseline session (day 1, completed between ~09:00-21:00) and six diurnal timepoints the subsequent day (day 2; diurnal timepoints 1–6, completed between ~08:00-00:00). The baseline session included self-report measures on demographics, medical diagnoses, psychiatric traits, psychiatric symptoms and behaviors, sleep, and chronotype. The diurnal timepoints, carried out approximately every three hours, involved ratings on psychiatric symptoms and behaviors engaged in in the previous hours. After these ratings, a brief cognitive test battery was completed (results not reported here). Data were time stamped and exact times were used for the analysis. Data collection took place in the autumn of 2021. The study was approved by the Swedish Ethical Review Authority (dnr: 2021-01695). Participants provided online informed consent during the baseline session.

### Psychiatric measures

#### Psychiatric traits and risk factors

Participants completed scales on a range of psychiatric traits and risk factors (referred to as “psychiatric traits” hereafter). See Table 2 for an overview of the scales and the Supplement for more detailed information on the scales.

**Table 2:** Overview of the scales measuring sleep and psychiatric traits.

Scale	Main outcome
Munich Chronotype Questionnaire (MCTQ)	Habitual sleep duration
Reduced Morningness-Eveningness Questionnaire (rMEQ)	Chronotype or circadian preference
Center for Epidemiologic Studies Depression Scale Revised Short Form (CESD-R 10)	Depression trait
Generalized Anxiety Disorder-7 (GAD-7)	Generalized anxiety trait
Altman Self-Rating Mania Scale (ASRMS)	Mania trait
Peters Delusions Inventory 21 (yes/no subscale) (PDI-21) and unusual experiences subscale of the Oxford-Liverpool Inventory of Feelings and Experiences (O-LIFE)	Delusional ideation trait
Difficulties in Emotion Regulation Scale-16 (DERS-16)	Emotion regulation difficulties trait
Autism Quotient-10 (AQ-10)	Autism trait
Health-relevant Personality Inventory (HP5i) impulsivity subscale	Impulsivity trait
Affective Lability Scale (ALS-18)	Emotional instability trait
Adult ADHD Self-Report Scale (ASRS)	ADHD trait
Obsessive Compulsive Inventory-Revised (OCI-R)	OCD trait
Eating Attitudes Test-26 (EAT-26) part B	Eating disorder trait
Apathy Evaluation Scale (AES)	Apathy trait
Liebowitz Social Anxiety Scale (LSAS)	Social anxiety trait

Scales were completed in the order as presented.

*ADHD* Attention Deficit Hyperactivity Disorder, *OCD* Obsessive Compulsive Disorder.

#### Psychiatric symptoms and behaviors

**Fig. 1:**
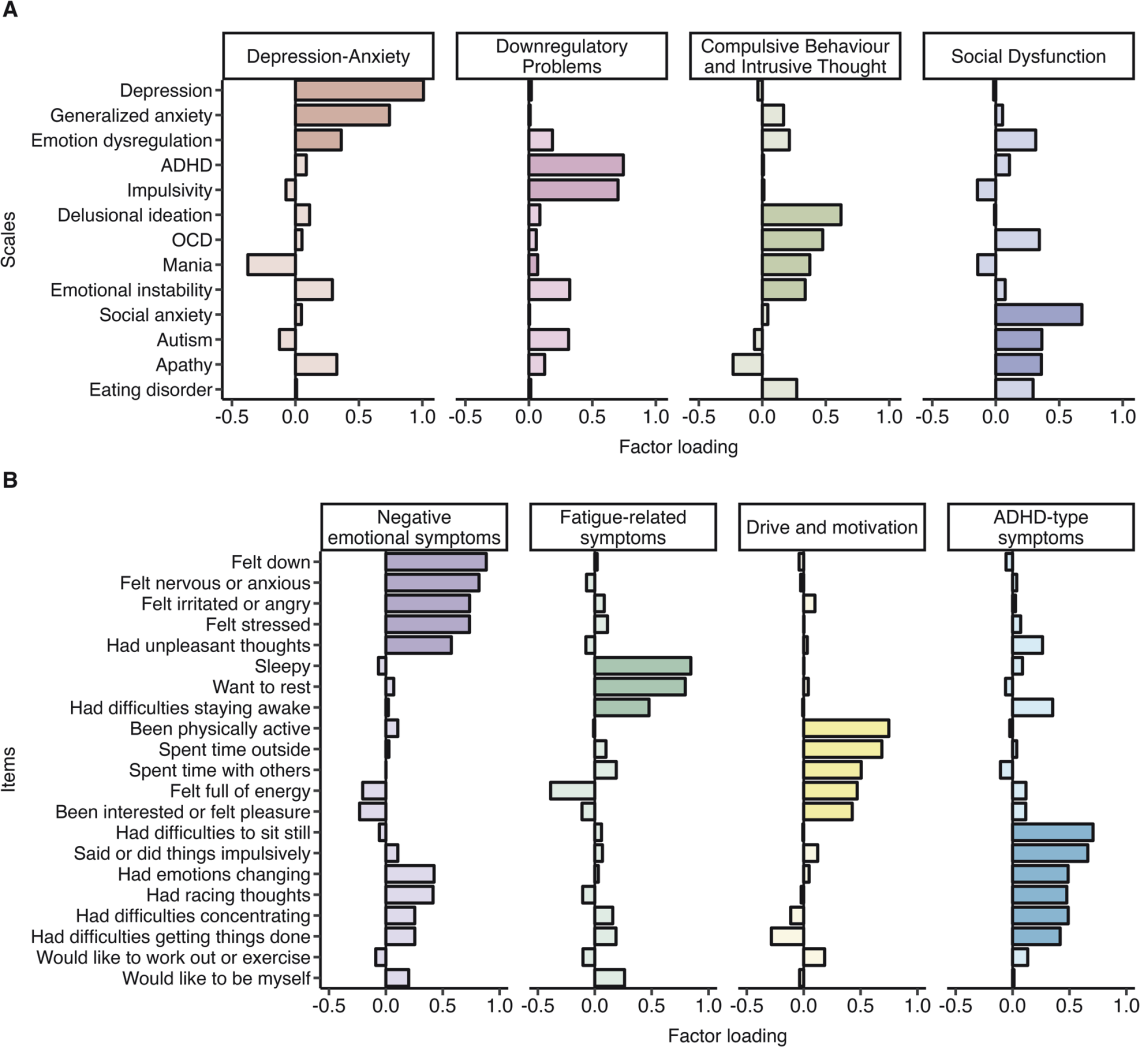
Factor loadings. Each panel shows the factor loadings for the constructs of the (**A**) transdiagnostic psychiatric traits and (**B**) psychiatric symptoms and behaviors. Higher factor loadings correspond to stronger loadings for the respective construct, with both positive and negative loadings. The scales are ordered according to strength of the factor loading when loading strongest on the respective construct. More saturated colored bars indicate the items that load ≥0.30 and load strongest on the respective construct. The items “would like to work out or exercise” and “would like to be by yourself” were not included in the factor calculations of (**B**) because these items did not significantly contribute to any of the factors (factor loading <0.30). The item “Would you like to be with a group of friends?” was removed because of insufficient sampling adequacy (Kaiser-Meyer-Olkin (KMO) < 0.60). Labeling of the factors was based on the scales/items that loaded most strongly on each respective construct.

Participants rated 22 items on psychiatric symptoms and behaviors (see Fig. 1b for the 21 items included in the final symptom constructs and Supplementary Table S2 for all 22 items). Items were rated from “1 = Not at all” to “9 = Very much/All the time” (rescored to 0 to 8 for analyses). The psychiatric symptoms and behaviors rated at baseline were used to retrieve the latent constructs of psychiatric symptoms and behaviors.

### Statistical analysis

#### Latent constructs psychiatric traits and psychiatric symptoms

Factor analysis was performed to obtain the latent constructs of the 13 psychiatric trait scales and psychiatric symptoms and behaviors (22 items) rated at baseline, using the *fa* function of the *Psych* R package [29]. The total score of each psychiatric trait scale was used as the sample size (*n* = 515) provided a too low subject-to-item ratio for factor analysis of all 231 items.

#### Relationships of chronotype with psychiatric traits and psychiatric trait constructs

Fixed effects regression analyses were used to assess the relationships of chronotype (continuous variable) with psychiatric traits and transdiagnostic psychiatric trait constructs. Predictors were Z-transformed before analysis to allow for comparison of coefficients. We also performed multinominal logistic regression models (using the *multinom* function of the *nnet* R package [30]) with chronotype as a categorical variable (see Supplement for results). In the preregistration we outlined reporting sleep profiles, but we decided to omit those to maintain clarity in this article. A subset of the participants (*n* = 138, 27%) inaccurately completed the rMEQ item “At what time in the evening do you feel tired and as a result in need of sleep?”, likely due to misunderstanding the question. This item was therefore omitted from the rMEQ calculation for these individuals. Their rMEQ scores were recalculated based on the remaining four items. See Supplement for more information.

#### Predicting diurnal variation in psychiatric symptom constructs based on an individual’s psychiatric profile and chronotype

Generalized additive mixed-effects models (GAMMs) were used to assess the extent and shape of any interaction between transdiagnostic psychiatric trait construct, time-of-day, and chronotype, allowing relationships to be non-linear. For each created GAMM, the best-fitting response distribution was assessed based on the Akaike information criterion (AIC) score comparisons. The significance of the fixed-effect predictors of interest was assessed with a forward stepwise model comparison approach, with model complexity increasing with one variable at a time. To increase reliability, double penalty shrinkage was applied to the predictors. This approach shrinks the effect of a given fixed-effect predictor toward zero if it does not meaningfully contribute to the model, thus reducing the risk for overfitting. Missing data (5430 out of 48,822 values, 11.1%) were imputed using the k-nearest neighbors (kNN) function of the VIM R package [31].

## Results

### Factor analysis of the psychiatric traits

Factor analysis of the psychiatric traits supported a 4-factor solution, labeled as “Depression-Anxiety” (D-A) (R^2^ = 0.19), “Downregulatory Problems” (DP) (R^2^ = 0.13), “Compulsive Behavior and Intrusive Thought” (CBIT) (R^2^ = 0.11), and “Social Dysfunction” (SD) (R^2^ = 0.10) (see Fig. 1a and Supplement for further details).

### Relationships between chronotype and psychiatric traits and constructs

#### Chronotype

Based on the rMEQ, 17.7% (*n* = 91) were categorized as morning-types (midpoint sleep free days (MSF) = 3:15), 59.4% (*n* = 306) as intermediate-types (MSF = 3:34), and 22.9% (*n* = 118) as evening-types (MSF = 3:55), with their average weekly sleep duration being 8h03, 7h54, 7h48, and last night’s sleep duration 7h34, 7h18 and 6h59, for the respective groups.

#### Psychiatric traits

**Fig. 2:**
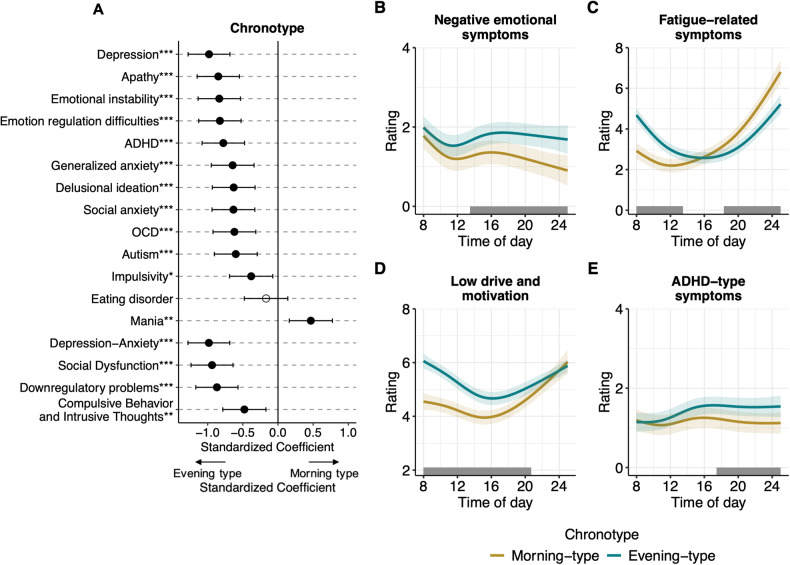
Relationships between chronotype, psychiatric traits and constructs, and diurnal symptom patterns. **A** Coefficient plot of the relationships of chronotype with the psychiatric traits and constructs and **B**–**E** diurnal patterns of symptom constructs for chronotype and psychiatric constructs. The coefficient plot shows how chronotype relates to 13 psychiatric traits and four transdiagnostic psychiatric trait constructs, ordered according to coefficient strength (0 = no relationship). A lower score of chronotype (reduced Morningness-Eveningness Questionnaire; rMEQ) on the x-axis indicates a stronger evening-type. Error bars represent 95% confidence intervals. Filled dots indicate statistically significant relationships, open dots indicate statistically non-significant relationships. Diurnal patterns of **B** negative emotional symptoms, **C** fatigue-related symptoms, **D** low drive and motivation, and **E** ADHD-type symptoms for morning- and evening-types. The Y-axes represent mean scores of the item ratings with each item having a minimum of 0 “not at all” and a maximum of 8 “Very much/All the time”. Note the different start and end points of the axes. The axis for **D** drive and motivation was reversed for ease of interpretation, with worse symptom levels (lower drive and motivation) being illustrated as higher on the scales. Error bands represent 95% pointwise confidence intervals. Gray bars show the timepoints where morning- and evening-types differ significantly. See Supplementary Fig. S4 for illustrations that also include intermediate-types. ***p < 0.001; **p < 0.01; *p < 0.05.

As shown in Fig. 2a, higher evening-type scores (lower rMEQ scores) were associated with (in decreasing order of strength): depression; apathy; emotional instability; emotion regulation difficulties; ADHD; generalized anxiety; delusional ideation; social anxiety; OCD; autism; impulsivity. Mania was the only trait associated with being a morning-type. There was no significant association of chronotype with eating disorder trait.

#### Transdiagnostic psychiatric trait constructs

Higher evening-type scores (lower rMEQ scores) were associated with all trait constructs, in decreasing strength: D-A; SD; DP; CBIT (Fig. 2a). See Supplement tables for more results including odds ratios (ORs) and age- and gender-corrected ORs.

### Factor analysis of the psychiatric symptoms and behaviors

The factor analysis of the psychiatric symptoms and behaviors supported a 4-factor solution (referred to as “symptom constructs” hereafter), labeled “Negative emotional symptoms” (R^2^ = 0.20), “Fatigue-related symptoms” (R^2^ = 0.10), “Drive and motivation” (R^2^ = 0.09), and “ADHD-type symptoms” (R^2^ = 0.13) (see Fig. 1b and Supplement for further details).

## Diurnal symptom patterns

**Fig. 3:**
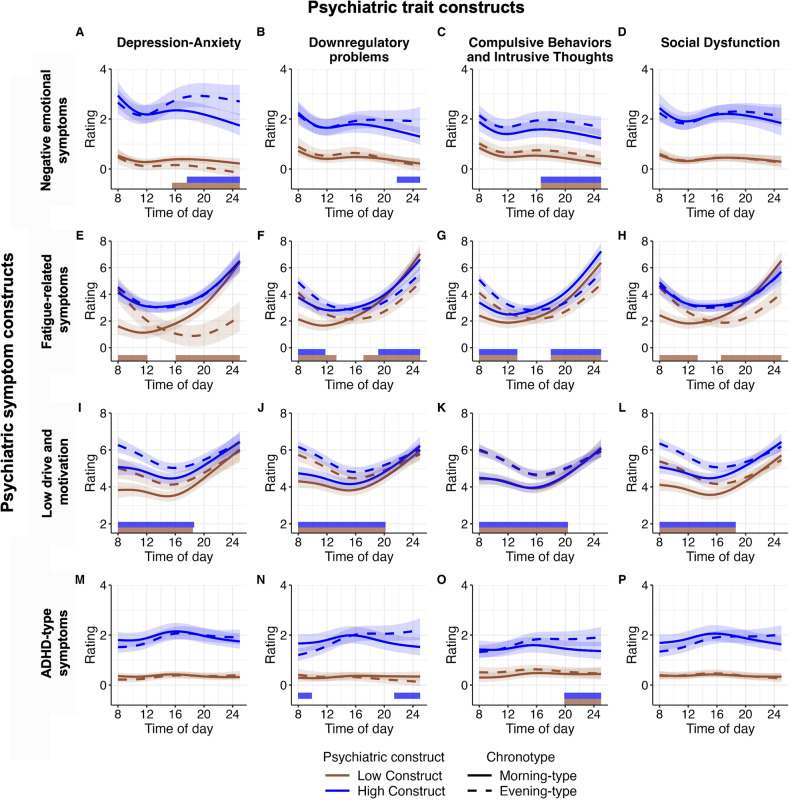
Diurnal patterns of psychiatric symptoms. Diurnal patterns of **A**–**D** negative emotional symptoms, **E**–**H** fatigue-related symptoms, **I**–**L** low drive and motivation, and **M**–**P** ADHD-type symptoms, separated by Depression-Anxiety construct (1^st^ column), Downregulatory Problems construct (2^nd^ column), Compulsive Behavior and Intrusive Thoughts construct (3^rd^ column), and Social Dysfunction construct (4^th^ column). All plots are stratified by psychiatric construct (Low and High) and chronotype (Morning-type (solid) vs Evening-type (dashed)). For visualization purposes only, the constructs are divided into low and high construct: Low Construct = mean(construct) – 1.5*standard deviation(construct). High Construct = mean(construct) + 1.5*standard deviation(construct). Therefore, the difference between the low and high construct is 3 standard deviations. Brown (low construct) and blue (high construct) bars show the timepoints where morning- and evening-types significantly differ. The Y-axes represent mean scores of the item ratings with each item having a minimum of 0 “not at all” and a maximum of 8 “Very much/All the time”. Error bands represent 95% pointwise confidence intervals. Note the different start and end points of the scales. The presented values are predicted GAMM values.

The results of the final GAMMs and all model comparisons are reported in Supplementary Tables S11–S46. Model predictions are visualized to ease interpretation (see Figs. 2 and 3 and Supplementary Figs. S4 and S5 for plots including intermediate chronotypes).

### Main effects of time-of-day and chronotype

As shown in Fig. 2b–e, all symptom constructs changed non-linearly across the day. Evening-types had more negative emotional symptoms and lower drive and motivation as compared to morning-types. Fatigue-related symptoms and ADHD-type symptoms did not significantly differ between morning- and evening-types. Intermediate-types had symptom levels in between morning- and evening-types for all symptom constructs (see Supplementary Fig. S4).

### Predictions of diurnal symptom construct patterns from chronotype

#### Negative emotional symptoms

Morning- and evening-types had highest levels of negative emotional symptoms in the early morning (~08:00). Morning-types showed a decreasing pattern from 16:00 until late, with lower levels than evening-types from ~13:00 and later (Fig. 2b).

#### Fatigue-related symptoms

Fatigue-related symptoms were highest in the early morning and late evening, with evening-types having a rhythm occurring a few hours later compared to morning-types. With respect to clock hours, morning-types had lower fatigue-related symptoms from the morning until the afternoon (~08:00-13:30) as compared to evening-types, but higher levels in the evening (~18:15 and later). Morning-types experienced the least fatigue ~12:15 and evening-types ~15:50 (Fig. 2c).

#### Low drive and motivation

Evening-types had lower drive and motivation than morning-types throughout most of the day, with largest differences ~08:00-20:15 (Fig. 2d).

#### ADHD-type symptoms

In the morning, both morning- and evening-types showed the lowest and comparable levels of ADHD-type symptoms. Evening-types experienced a rising pattern until ~16:00, reaching stable and more severe levels than morning-types from ~17:30 and onwards (Fig. 2e).

These analyses were repeated after excluding individuals who reported having a psychiatric disorder (see Supplementary Fig. S6), and resulted in similar findings.

### Predictions of diurnal symptom construct patterns from chronotype and transdiagnostic trait constructs

The psychiatric trait constructs predicted worse levels in all symptom constructs (i.e., negative emotional symptoms, fatigue-related symptoms, low drive and motivation, and ADHD-type symptoms), except for low drive and motivation in the CBIT construct (see Fig. 3).

### Negative emotional symptoms

The elevation in negative emotional symptoms in the early morning was most pronounced in those higher in psychiatric construct, similarly for morning- and evening-types. Morning-types high in D-A, DP, or CBIT trait construct experienced an alleviation of negative emotional symptoms in the evening, while evening-types increased in negative emotional symptoms in the afternoon (particularly those high in D-A) and continued to have elevated symptoms until late.

### Fatigue-related symptoms

Among individuals low in the transdiagnostic psychiatric trait constructs, fatigue followed the synchrony effect, i.e., worse fatigue at times that are converse to someone’s optimal time-of-day according to their chronotype. This was similarly observed for those with high levels of DP or CBIT, but not D-A or SD.

### Low drive and motivation

The difference in drive and motivation between evening- and morning-types was evident across all transdiagnostic psychiatric trait constructs, regardless of their level. These differences, marked by lower drive and motivation in evening-types, were particularly pronounced during the morning hours (~08:00-12:00). In the morning, evening-types high in D-A or SD displayed the lowest drive and motivation compared to morning-types and their low psychiatric trait construct counterparts. However, by the late evening, drive and motivation levels became similar across all groups.

### ADHD-type symptoms

Individuals low in psychiatric trait constructs showed generally stable and low-level ADHD-type symptoms across the day. In contrast, evening-types high in psychiatric trait construct varied more across the day. Morning-types high in psychiatric trait constructs had stable ADHD-type symptom levels throughout the day with slightly elevated symptoms ~16:00, and morning-types high in DP having more symptoms than evening-types from ~08:00-10:00. Evening-types showed increasing symptom levels throughout the day, with higher levels than morning-types in the evening for DP and CBIT (from ~21:30 for DP and ~20:00 for CBIT).

## Discussion

The primary goal of the current preregistered study was to establish a better understanding of the relationships between chronotype (i.e., diurnal preference) and psychiatric traits, and to determine how well an individual’s combination of psychiatric traits and chronotype predict diurnal variation in psychiatric symptoms and behaviors.

Our dimensional approach revealed that 11 of the 13 psychiatric traits were related to being an evening-type. These were, in order of decreasing strength: depression; apathy; emotional instability; emotion regulation difficulties; ADHD; generalized anxiety; delusional ideation; social anxiety; OCD; autism; impulsivity traits. Mania was related to being a morning-type, whilst eating disorder trait was not related to chronotype. The psychiatric trait scales loaded onto four factors, with all being associated with being an evening-type. These results confirm robust associations between the evening chronotype and psychiatric phenotypes more broadly than described previously [26], also including traits relating to delusional ideation, OCD, and ADHD, that have previously received limited attention. The presence of relationships between chronotype and psychiatric phenotypes across the sub-clinical range emphasizes the importance to investigate the involvement of chronotype in the etiology of psychiatric disorders.

The 22 psychiatric symptoms and behaviors rated across the day loaded onto four state factors, relating to *negative emotional symptoms*, *fatigue-related symptoms*, *low* *drive and motivation*, and *ADHD-type symptoms*. While individuals with worse levels of psychiatric traits generally experienced worse daytime levels of symptom constructs, it was primarily chronotype that predicted how symptom constructs varied across the day. Fatigue-related symptoms showed the most pronounced diurnal pattern, peaking in the morning and late evening. In line with the synchrony effect [28], the timing of fatigue (but not the other symptom constructs) was shifted depending on chronotype: evening-types, as compared to morning-types, reported higher levels of fatigue in the morning and vice versa in the evening, particularly in individuals with low psychiatric trait levels. This is consistent with evening-types having to rise earlier in their circadian phase than morning-types, and that morning-types are awake later into their circadian phase [32].

Drive and motivation changed substantially across the day and evening-types reported lower levels across most of the day, particularly in the morning. The fact that evening-types suffered from worse drive and motivation all day and into the evening was unexpected. These low levels of drive and motivation in the morning may represent a key problem, contributing to problems rising from bed and hindering engagement in morning activities that include light exposure, which may further consolidate their late chronotype. Both animal and human research indicate diurnal variation in the motivation-relevant dopamine pathway [33–35], but there is still a poor understanding regarding the shape of diurnal patterns of motivation-related behaviors and their subcomponents, see refs for differences in peaks [18, 33, 34, 36–40]. This highlights the need to investigate the role of chronotype and diurnal changes in motivation for symptom development. Likewise, there is no consensus with respect to the diurnal pattern of negative affect (e.g., refs. [18, 38–41]). Our sample showed small diurnal variation in negative emotional symptoms, with higher levels in the morning, and improvements for morning-types in the evening hours.

Our data suggest that evening-types with more severe psychiatric trait levels, i.e., individuals at the disadvantageous ends of the psychiatric trait scales, show a rising pattern of ADHD-type symptoms across the day. The general minor diurnal pattern on the group level is somewhat surprising. It is, however, possible that variability in ADHD-symptoms is more strongly regulated by contextual and homeostatic factors such as insufficient sleep [42]. Further steps include characterizing how separate ADHD-type symptoms vary and relate to specific traits.

Evening-types were worse off for most symptoms: in the morning hours for fatigue-related symptoms and low drive and motivation and in the evening hours for negative emotional symptoms and ADHD-type symptoms. Potential reasons for the worse morning symptom levels are that evening-types have accumulated sleep debt [43] and/or rise early in their circadian phase [44]. The accumulating sleep pressure and an early rise in the circadian phase may also have downstream effects on cognitive and emotional functioning later in the day, contributing to negative emotional symptoms and ADHD-type symptoms. Light exposure close to biological dusk entrains someone’s circadian phase to the natural light-dark cycle [44]. Interventions such as early morning light exposure, physical activity, caffeine intake or sleep cycle adjustment therapy or medication (e.g., melatonin) may potentially have acute positive mental effects (e.g., reduce fatigue and stimulate motivation), simultaneously phase advance rhythms, and potentially improve sleep in evening chronotypes [45]. Future intervention studies are needed to assess the clinical validity of these findings. The current findings also suggest that diurnal patterns of psychiatric symptoms could be used to better manage workload to meet the optimal time-of-day for the individual. This alignment may mitigate work-related stress and potentially prevent sick leave.

A primary limitation of the current study is the limited control of the participants’ surroundings during ratings. However, participants were instructed to find an undisturbed space and attention checks were included to reduce the influence of external factors. In line with the RDoC, we chose a dimensional approach measuring psychiatric traits along a continuum from low-level symptoms to symptoms meeting diagnostic criteria [27]. This approach extends the relevance of our results to a general population sample. Despite a dimensional approach and high number of individuals with possible clinical levels of self-reported psychiatric traits (potentially due to the timing of data collection (autumn 2021) during the COVID-19 pandemic when psychological well-being decreased in the general population [46]), it remains to be determined whether results generalize to clinically confirmed disorders. Furthermore, the rMEQ was used to estimate chronotype, but alternative methods could be assessed, such as melatonin phase or actual sleep timing (that are nevertheless related to rMEQ). Strengths of the study include the preregistration, sampling of a large number of timepoints which enabled us to model diurnal effects between ~08:00-00:00, and the inclusion of a wide range of psychiatric traits. Moreover, the assessments in the individual’s natural environment potentially removed barriers for those unable to visit testing laboratories due to mental or physical limitations, thereby improving the study’s ecological validity.

In conclusion, these data show that the evening chronotype represents a transdiagnostic factor shared by most subclinical psychiatric phenotypes. The prominent differences in drive and motivation across the day and between chronotypes as well as the evening worsening of symptoms in evening-types reveals an important avenue for approaches to promote mental health. Together these findings highlight the importance of considering time-of-day and chronotype when designing treatments to optimize health and functioning as well as for research designs.

### Supplementary information

Supplemental Material

## Data Availability

Data and code are available upon request.
