# Supplementary material for: The rhythm of mental health: the relationship of chronotype with psychiatric trait dimensions and diurnal variation in psychiatric symptoms
Source: Transl Psychiatry. 2024 Jun 4;14:237. doi: 10.1038/s41398-024-02943-7 (PMC11150537; doi:10.1038/s41398-024-02943-7)
Supplement: Supplementary file 1 — Supplemental Material [file 41398_2024_2943_MOESM1_ESM.pdf]

Supplementary Information

	Page
**Supplementary Methods**	
Recruitment, drop-out, quality checks, and exclusion	2
Study design	2
Sleep measures and psychiatric trait and risk factor scales	2-4
Statistical approach	4-5
**Supplementary Results**	
Factor analysis results	5
Relationships of psychiatric traits and transdiagnostic psychiatric trait constructs with chronotype	6
**Supplementary Figures**	
Figure S1: Density plots depicting the distribution of observed and imputed data	7
Figure S2: Correlation matrix psychiatric traits	8
Figure S3: Frequency plots psychiatric traits and age	9
Figure S4: Diurnal patterns of symptom constructs with intermediate-types	10
Figure S5: Diurnal patterns of symptom constructs by transdiagnostic psychiatric trait constructs with intermediate-types	11-12
Figure S6: Diurnal patterns of symptom constructs without individuals with a self-reported psychiatric diagnosis	13
Figure S7: Scree plots	14
Figure S8: Frequency plot of reduced Morningness-Eveningness Questionnaire	15
**Supplementary Tables**	
Table S1: Number of completions per time bin	16
Table S2: Psychiatric symptoms and behaviors items	17
Table S3: Mean score, range, and possible clinical cut-off for each psychiatric trait scale	18
Table S4: Factor loadings of the psychiatric trait scales	19
Table S5: Transdiagnostic psychiatric trait construct correlations	20
Table S6: Symptom construct correlations	21
Table S7: Coefficients for the relationships between chronotype and psychiatric traits and transdiagnostic psychiatric trait construct (univariable and multivariable models)	22
Table S8: Odds ratios for the relationships between chronotype and psychiatric traits and transdiagnostic psychiatric trait construct	23
Table S9: Age- and gender-correct odds ratios for the relationships between chronotype and psychiatric traits and transdiagnostic psychiatric trait construct	24-25
Table S10: Factor loadings of the psychiatric symptoms and behaviors	26
Table S11-S14: GAMM results for predicting changes in symptom constructs from chronotype	27-30
Table S15-S30: Model comparisons: Akaike information criterion (AIC) scores	31-46
Table S31-S46: GAMM results for predicting changes in symptom constructs from chronotype and transdiagnostic psychiatric trait construct	47-62
**SI References**	63-64

SUPPLEMENTARY METHODS

Recruitment

Sixty-five participants were recruited to assess feasibility and dropout rates. Participants in this batch completed a baseline session and five timepoints (separated by about four hours) between ~08:00-00:00. The subsequent data collections included six timepoints (separated by about three hours each).

Drop-out, quality checks, and exclusion

The baseline session was started by 747 individuals of which 142 were duplicates (i.e., started the study twice of which the first or the most complete entry was kept). Data for the factor analysis of the psychiatric symptoms and behaviors was available for 605 individuals, and 515 individuals completed the psychiatric scales in the baseline session (90 stopped the baseline session prematurely). As per preregistration, to control for possible inattention during the baseline session, two attention checks and one honesty check were included [1]: “Please rate the response alternative 'agree’) for this question”; “Please answer 100”; “Have you been completely honest in your answers?”. Baseline data of participants who failed more than one attention/quality check (*n* = 6) were excluded. The next day, 410 contributed at least two diurnal timepoints and for which baseline data were available. The median number of completed timepoints was six.

Study design

Data collection for this study occurred on weekdays only. Participants were divided into two groups for the diurnal timepoints. All were instructed to start the first diurnal timepoint of day 2 within the time window of 08:00-09:00. This first start time was self-chosen to reduce potential interference with habitual sleep duration/wake time. This morning timepoint was followed by five timepoints with start times that were separated by three hours. Group 1 was instructed to start the subsequent timepoints at 10:00, 13:00, 16:00, 19:00, and 22:00, and group 2 was instructed to start the subsequent timepoints at 11:00, 14:00, 17:00, 20:00, and 23:00. The one-hour shift between the groups provides data for more timepoints across the day. All data were time stamped and the time stamp was used for the analysis. Participants were encouraged to start each completion at the instructed time; however, they could complete each respective timepoint also after the start times in order to maximize the number of available datapoints. After the psychiatric symptoms and behaviors ratings, a brief cognitive test battery was completed (results not reported here). Each diurnal timepoint took about 15 minutes to complete.

Sleep measures and psychiatric trait and risk factor scales

Sleep measures

MCTQ. The Munich Chronotype Questionnaire (MCTQ) [2] is a scale that estimates an individual’s phase of entrainment on work and work-free days. The MCTQ was used to collect information about average weekly sleep duration and the midpoint of sleep, i.e., midpoint between sleep onset and offset on free days.

rMEQ. Circadian preference, or chronotype, was assessed using the reduced Morningness-Eveningness Questionnaire (rMEQ) [3]. The rMEQ measures the preferred times of day for certain activities. A subset of the participants (n = 138, 27%) inaccurately completed the rMEQ item “At what time in the evening do you feel tired and as a result in need of sleep?”, likely due to misunderstanding the question. This item was therefore omitted from the rMEQ calculation for

these individuals. Their rMEQ scores were recalculated based on the remaining four items (range 3-21) and aligned with the 4-25 range, consistent with the possible range of the 5-item rMEQ). See Figure S8 for a frequency plot comparing the distribution of the rescored 4-item scores and the original 5-item scores.

Last night's sleep duration. Last night's sleep duration was calculated from bedtime and time to fall asleep the night before the diurnal timepoints, and wake time the morning of the test day.

Psychiatric trait and risk factor scales

The following validated measures were used to assess psychiatric traits. In all scales, a higher score indicates a higher level of the trait. Scales that asked for symptoms over the past week or two weeks were adapted to ask for symptoms over the past month (CESD-R 10, GAD-7, ASRMS, ALS-18, OCI-R). See Figure S2 for a heatmap of the correlations between the psychiatric trait scales, Figure S3 for frequency plots of the scales, and Table S3 for mean scores and percentage of participants scoring above suggested clinical cutoff scores.

CESD-R 10. The Center for Epidemiologic Studies Depression Scale Revised Short Form (CESD-R 10) [4] is a 10-item scale designed to measure depression in the general population. Two items are reverse scored. A total score is calculated by summing the ratings: 0 "rarely or none of the time"; 1 "some or a little of the time"; 2 "occasionally or a moderate amount of time"; 3 "almost all of the time".

GAD-7. The Generalized Anxiety Disorder-7 (GAD-7) [5] is a 7-item scale designed to identify probable cases of GAD. The ratings (0, 1, 2, or 3) are added to retrieve a total score. Total scores of 5, 10, and 15 are taken as the cut-off points for mild, moderate, and severe anxiety, respectively.

ASRMS. The 5-item Altman Self-Rating Mania Scale (ASRMS) [6] is designed to assess the presence and severity of manic symptoms. A total score is calculated by summing the item ratings (0, 1, 2, 3, 4). A cutoff score of six or higher serves as an indication for further assessment of (hypo)mania.

PDI-21 and O-LIFE subscale. Delusional ideation was assessed using the yes/no subscale of the Peters Delusions Inventory 21 (PDI-21) [7] and the unusual experiences subscale of the Oxford-Liverpool Inventory of Feelings and Experiences (O-LIFE) [8]. The PDI-21 was designed to measure schizotypal traits in the general population. The combined PDI-21 and O-LIFE unusual experiences subscale consisted of 33 yes/no items, asking for occurrence of experiences during their lifetime, such as "do you ever feel that there is a conspiracy against you?" and "are your thoughts sometimes so strong that you can almost hear them?". Delusional ideation was scored by summing the count of all endorsements ("yes" responses).

DERS-16. The difficulties in emotion regulation scale (DERS-16) [9] is a 16-item scale designed to measure overall emotion regulation difficulties. The total score is obtained by summing the ratings (1, 2, 3, 4, 5) of each item.

AQ-10. The Autism Quotient-10 (AQ-10) [10] is a scale to measure the extent of autistic traits in adults. Items are rated on a 4-point scale. The items are scored with 0 or 1. The answer options "definitely agree" and "slightly agree" are scored as 1 for item 1, 7, 8, and 10, while the other items are scored as 1 for "definitely disagree" and "slightly disagree". Item ratings are summed to receive a total score. A cut-off score of six or higher indicates a significant number of autistic traits.

Impulsivity subscale of HP5i. The 20-item Health-relevant Personality Inventory (HP5i) [11] measures five health-relevant personality traits. The impulsivity subscale was used for this study.

Items are rated on a 4-point scale, ranging from "does not apply at all" to "applies completely". The impulsivity score is calculated by averaging four items.

ALS-18. The Affective Lability Scale (ALS-18) [12] is an 18-item scale designed to measure rapid shifts in outward emotional expressions, also referred to as emotional instability. The items are rated on a 4-point scale from 0 to 3 ("very uncharacteristic of me" to "very characteristic of me") and a total score is calculated by summing the ratings.

ASRS. The Adult ADHD Self-Report Scale (ASRS) [13] was completed to measure ADHD traits. The ASRS consists of 18 items about frequency of DSM-IV Criterion A symptoms of adult ADHD. The "ASRS part A" consists of six selected items to optimize concordance with the clinical classification. Items are rated on a 5-point scale, with ASRS part A scored as 0 or 1, based on specific criteria for each item. ASRS Part A was used to determine possible clinically significant levels, as per scale guideline. No total score is utilized for the remaining 12 items. However, we have scored the ASRS as follows for the main analyses: never (1), rarely (2), sometimes (3), often (4), very often (5). The total score is calculated by summing these scores.

OCI-R. The Obsessive Compulsive Inventory-Revised (OCI-R) [14] is an 18-item scale to measure the severity and type of OCD symptoms present, rated on a 5-point scale from 0 ("not at all") to 4 ("extremely"). The total score is calculated by summing the item ratings.

EAT-26 part B. Part B of the Eating Attitudes Test (EAT-26) [15] was completed to measures eating disorder trait. This 26-item scale contains items on eating-related attitudes, feelings, and behaviors, that are rated on a 6-point scale from "always" to "never", with "always" scored as 3, "usually" as 2, "often" as 1, and "sometimes", "rarely" and "never" as 0. One item is reverse scored. The total score is calculated by summing the ratings.

AES. The Apathy Evaluation Scale (AES) [16] measures behavioral, cognitive, and emotional concomitants of deficits in goal-directed behavior that reflect apathy. This 18-item scale is scored on a 4-point scale, from 1 ("not at all") to 4 ("a lot"). Three items are reverse scored and the total score is calculated by summing all item ratings.

LSAS. The Liebowitz Social Anxiety Scale (LSAS) [17,18] assesses fear/anxiety and avoidance of common situations. The 24 items are rated twice: once on fear or anxiety, from 0 ("none") to 3 ("severe"), and once on avoidance, from 0 ("never (0%)") to 3 ("usually (67-100%)"). The total score is calculated by summing the ratings of both subscales.

Statistical approach

Data imputation

K-nearest neighbors was used to impute missing data. To incorporate the temporal patterns in the data, time of day information was used as a distance variable. In other words, observations with a similar time of day were given higher weights in the imputation process. The nine nearest neighbors were used for imputing missing values. See Figure S1 for density plots of imputed (red) versus original (blue) data.

Latent constructs psychiatric traits and psychiatric symptoms

Gillan et al. [19] identified a 3-factor solution using items from nine psychiatric trait scales. These factors were labelled as *anxious-depression, compulsive behavior and intrusive thought*, and *social withdrawal*. As per preregistration (accessible at https://doi.org/10.17605/OSF.IO/8TM5U), we expected similar constructs. However, due to the inclusion of additional scales such as ADHD,

autism, mania, emotional instability, a 4-factor structure was expected. The decision regarding the number of factors was based on a combination of Horn's parallel analysis, Cattell's criterion, and theory. An oblique rotation technique (oblimin) was used. Estimated factor scores for the psychiatric traits were derived using Thurstone's least squares regression method. To facilitate interpretation and to preserve the variation in the original data, the psychiatric symptoms and behaviors factor scores were computed through averaging items by factor. All symptom ratings were on the same metrics. In case where items cross-loaded, they were included in the calculation of the factor on which they loaded most strongly. Maximum likelihood was used to estimate model parameters.

Relationships of chronotype with psychiatric traits and psychiatric trait constructs
We took a "soft" transdiagnostic approach [20] in which we obtained latent constructs cutting across a range of common psychiatric traits while also examining relationships between chronotype and individual psychiatric traits, preserving the underlying diagnostic classifications.

Predicting diurnal variation in psychiatric symptom constructs from an individual's psychiatric profile and chronotype
Gaussian distributions were used for the GAMM analyses. In cases where the response data were zero inflated (i.e., low frequency of positive responses), data were log transformed to avoid impossible predictions, such as symptom predictions below zero. Log transformed data were back-transformed for visualization purposes. Fixed-effect predictors were added in the following order: time-of-day (represented continuously from ~08:00-00:00), transdiagnostic psychiatric trait construct score, chronotype (categorized into morning, intermediate, evening), interactions of chronotype x time-of-day (to assess sensitivity to suboptimal timings), interactions of chronotype x transdiagnostic psychiatric trait construct x time-of-day (to assess whether those higher in the transdiagnostic psychiatric trait construct are more sensitive to suboptimal timings). A decrease in AIC of two or more was used as criterion for supporting the updated model. Since all the GAMMs converted we did not carry out any mixed effects models on how chronotype predicts diurnal variation, as stated in the pre-registration.

SUPPLEMENTARY RESULTS

Factor analysis results
Psychiatric trait constructs*.* The overall Kaiser-Meyer-Olkin (KMO) was 0.88, verifying good sampling adequacy for the analysis. All psychiatric traits had a KMO ≥ 0.68. Tucker Lewis fit index was 0.96. The scree plot can be found in Figure S7a. See Table S5 for correlations between the transdiagnostic psychiatric trait constructs.
Symptom constructs. The overall KMO was 0.91, suggesting good sampling adequacy. One item had a KMO value <0.60 ("Would you like to be with a group of friends?") and was therefore removed. Items with cross-loadings were included in the factor on which it loaded highest. Tucker Lewis fit index of factoring reliability was 0.88. The scree plot can be found in Figure S7b. See Table S6 for correlations between the symptom constructs.

Relationships of psychiatric traits and transdiagnostic psychiatric trait constructs with chronotype – multinominal logistic regression models

Psychiatric traits. Being an evening-type, as compared to being a morning-type, was associated with increased odds of scoring higher on (decreasing strength): depression; ADHD; emotional instability; apathy; emotion regulation difficulties; delusional ideation; autism; generalized anxiety; social anxiety; OCD; impulsivity traits. Being a morning-type, versus being an evening-type, was associated with increased odds for mania (non-significantly, 95% CI [0.58, 1.01]). There was no significant association of chronotype with eating disorder trait. See Table S8 for the odds ratios (OR) with 95% confidence intervals (CI) and Table S9 for age and gender corrected OR and corresponding 95% CI.

Transdiagnostic psychiatric trait constructs. Depression-anxiety, social dysfunction, downregulatory problems, and compulsive behavior and intrusive thought were all associated with increased odds of being an evening-type versus morning-type.

SUPPLEMENTARY FIGURES

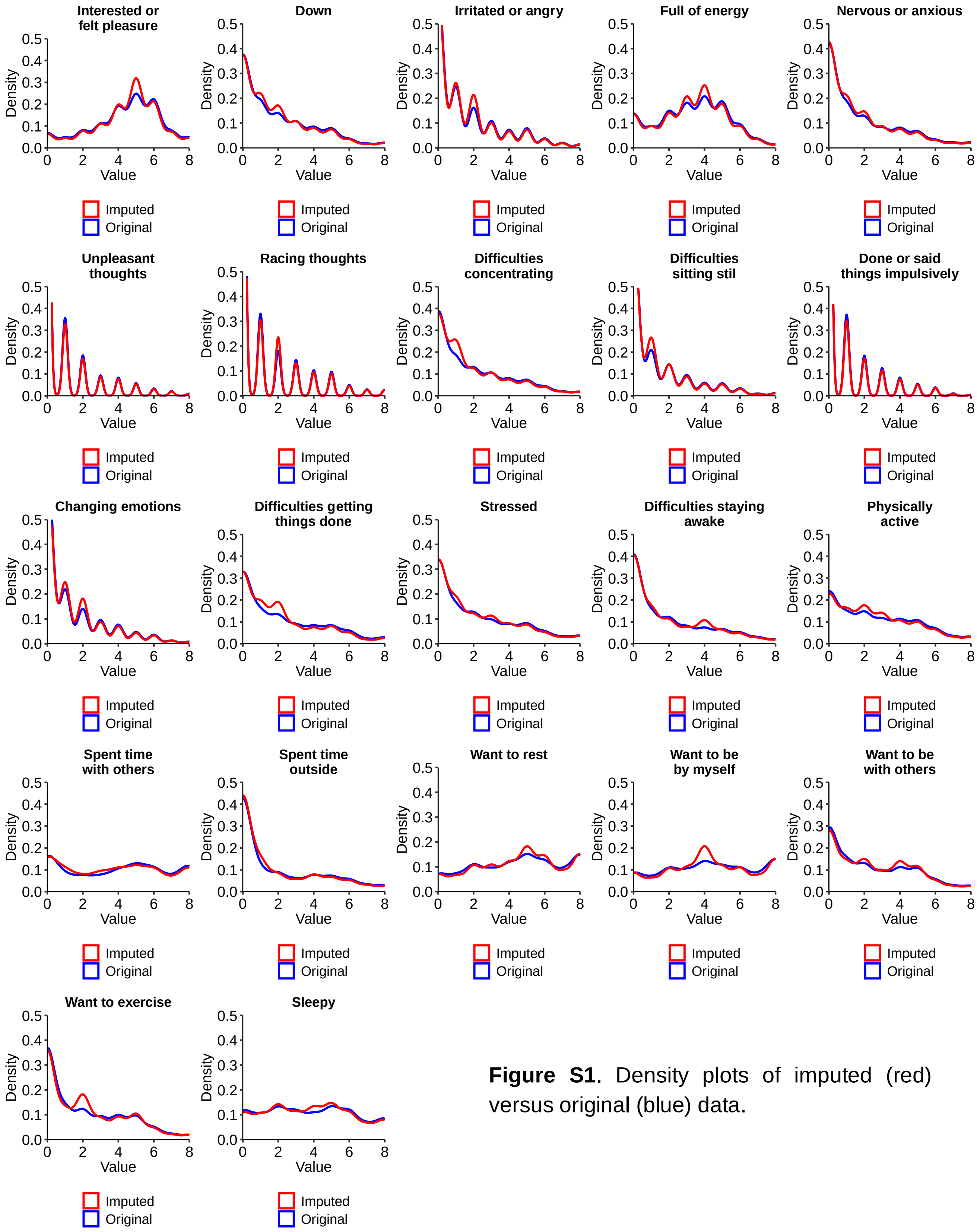

Figure S1. Density plots of imputed (red) versus original (blue) data.

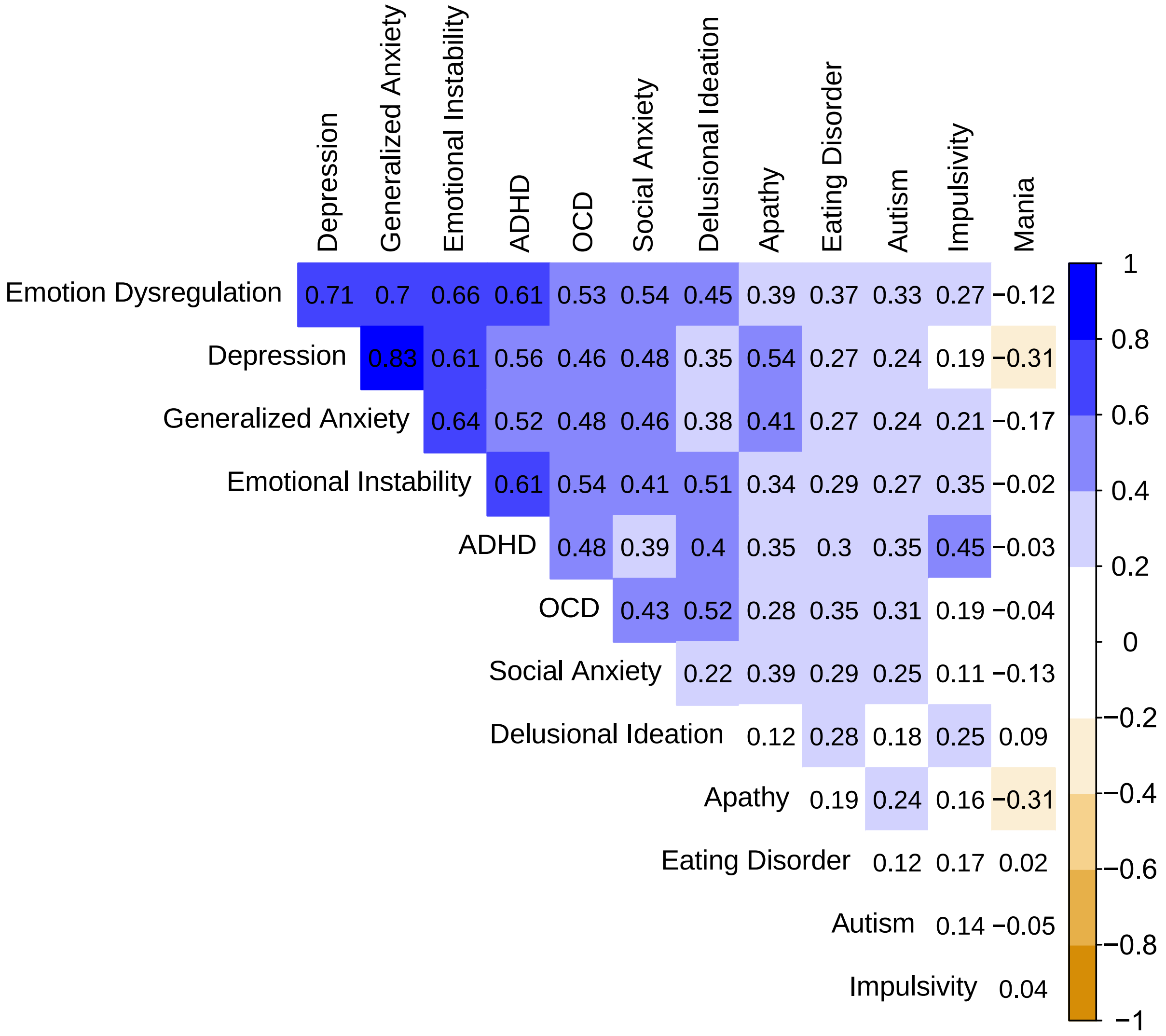

Figure S2. Correlation matrix showing Pearson correlation coefficients between all psychiatric trait and risk factor scales. For all measures, a higher score indicates a worse trait/risk factor level.

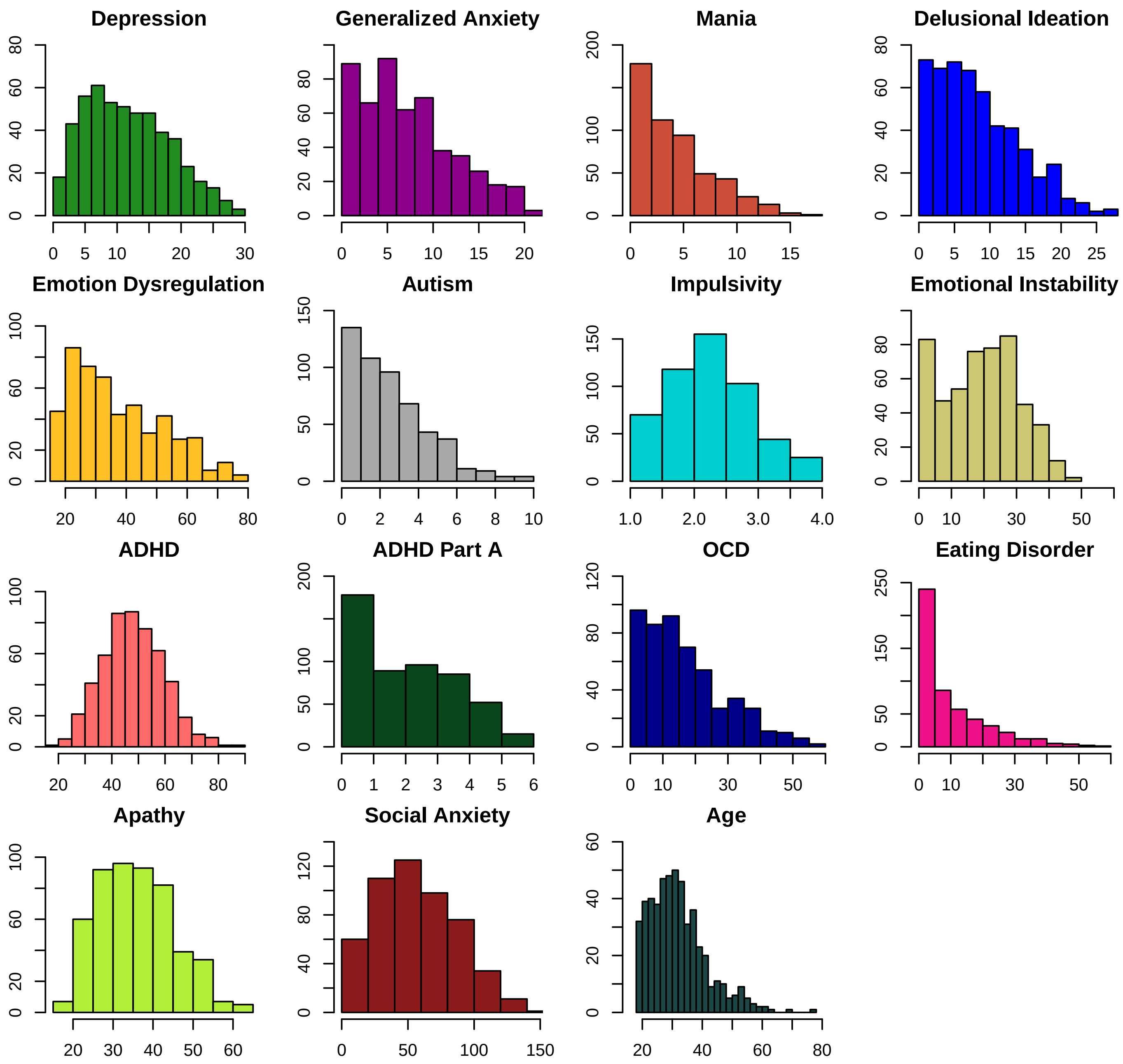

Figure S3. Frequency plots of the scores of the psychiatric trait and risk factor scales and age. See possible range of scale scores in Table S3 (age range 18-77).

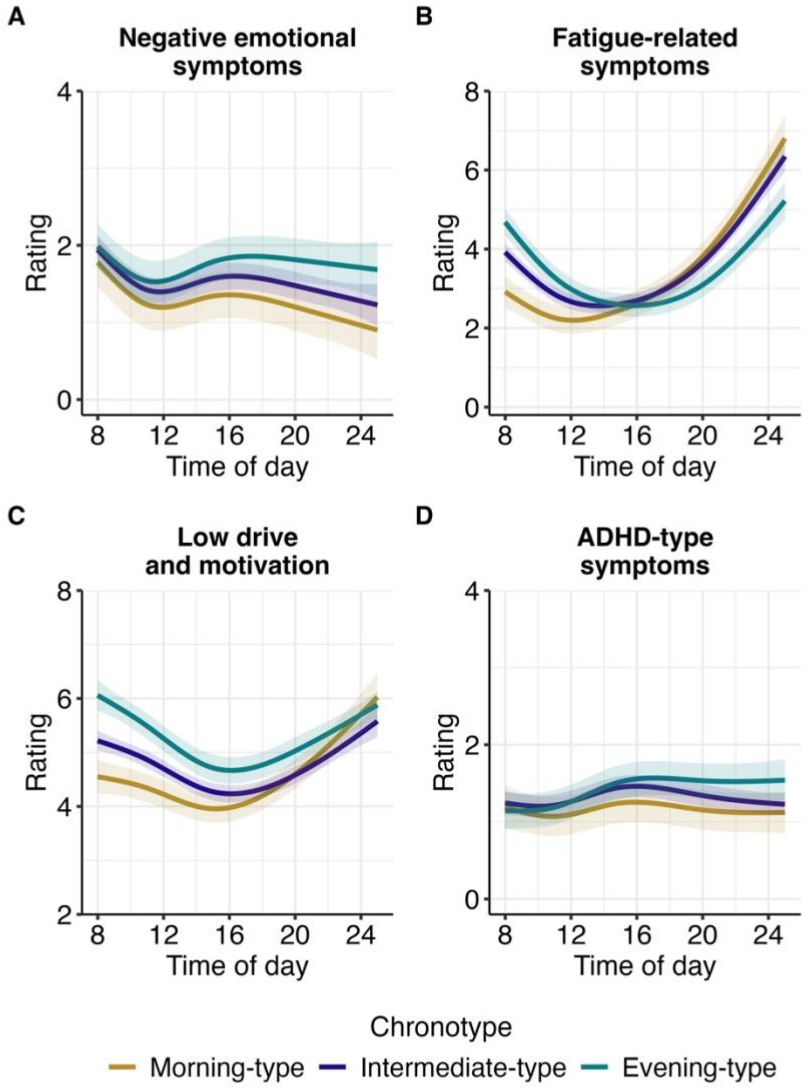

Figure S4. Diurnal effects of (A) negative emotional symptoms, (B) fatigue-related symptoms, (C) low drive and motivation, and (D) ADHD-type symptoms, stratified by chronotype (Morning-type, Intermediate-type, Evening-type). Error bands represent 95% pointwise confidence intervals.

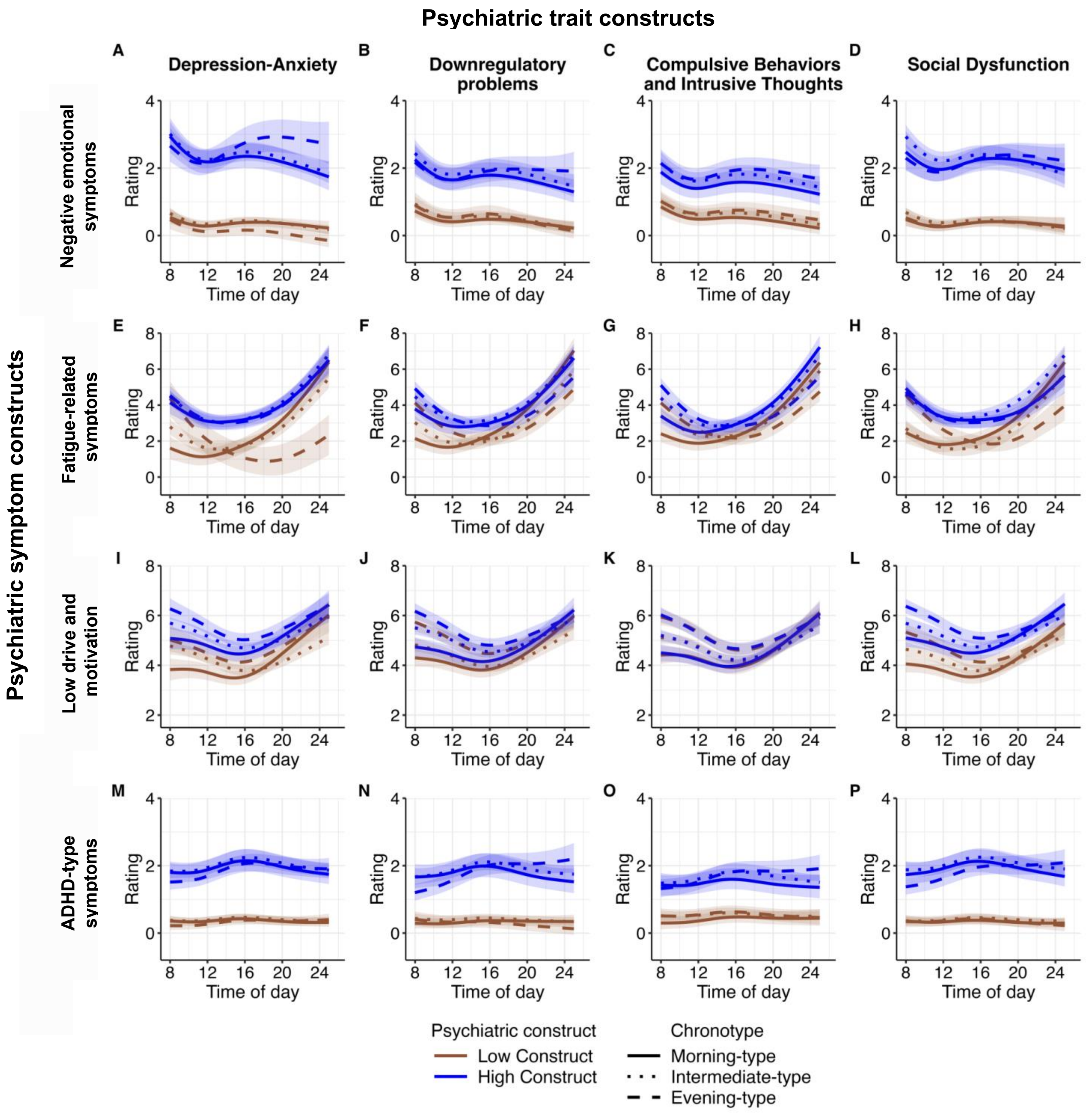

Figure S5. Diurnal patterns of psychiatric symptoms, including morning- intermediate- and evening chronotypes (A-D) negative emotional symptoms, (E-H) fatigue-related symptoms, (I-L) low drive and motivation, and (M-P) ADHD-type symptoms All plots are stratified by psychiatric construct (low and high) and chronotype (Morning-type (solid), Intermediate-type (dotted), and Evening-type (dashed)). For visualization purposes only, the constructs are divided into low and

high construct: Low construct = mean(construct) – 1.5*standard deviation(construct). High construct = mean(construct) + 1.5*standard deviation(construct). Therefore, the difference between the low and high construct is 3 standard deviations. The Y-axes represent mean scores of the item ratings with each item having a minimum of 0 “not at all” and a maximum of 8 “Very much / All the time”. Error bands represent 95% pointwise confidence intervals. Note the different start and end points of the scales. The presented values are predicted GAMM responses.

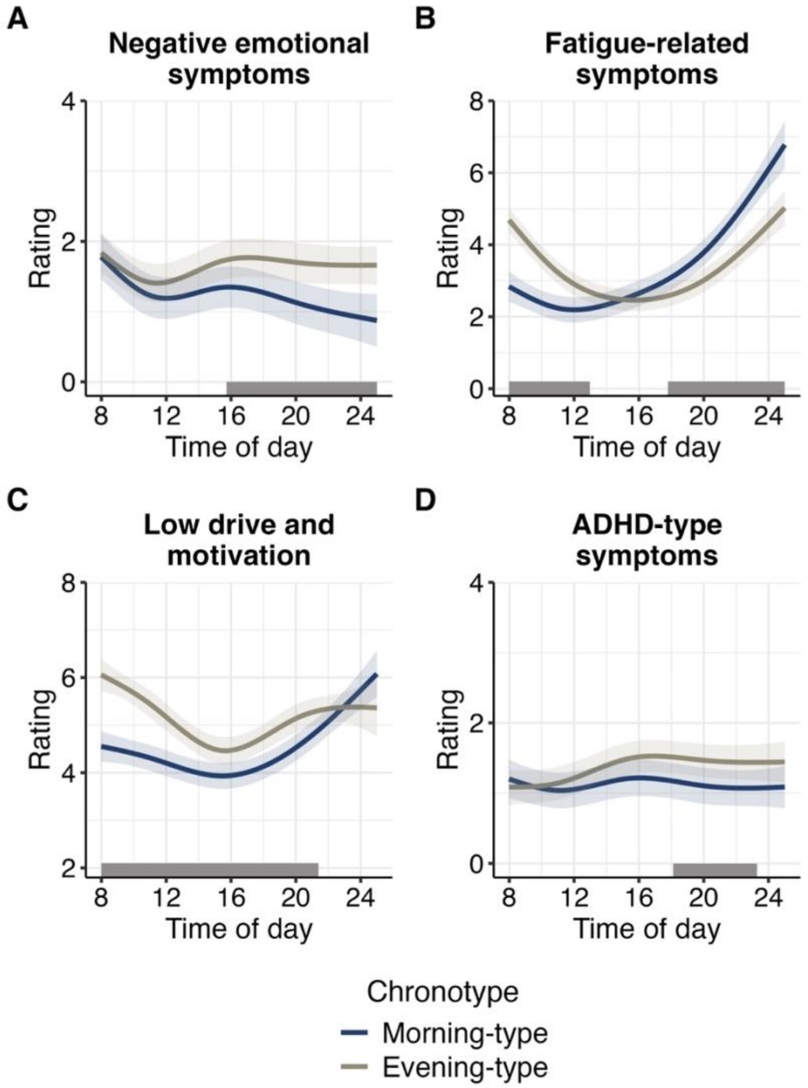

Figure S6. Time-of-day patterns of symptom constructs, stratified by chronotype (Morning-type and Evening-type). Analyses excluded individuals with a self-report psychiatric diagnosis (*n* = 21). Gray bars indicate timepoints where morning- and evening-types significantly differ. Error bands represent 95% pointwise confidence intervals.

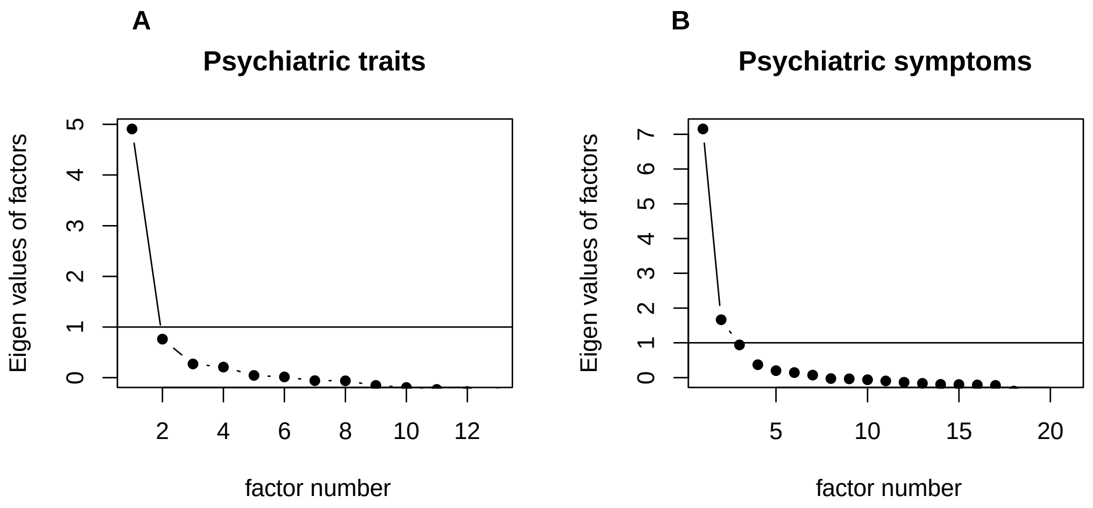

Figure S7. Scree plots of eigenvalues for (A) psychiatric traits and (B) psychiatric symptoms and behaviors. The final number of factors was based on a combination of Horn's parallel analysis, Cattell's criterion, and theory. This led to four factors for both A and B.

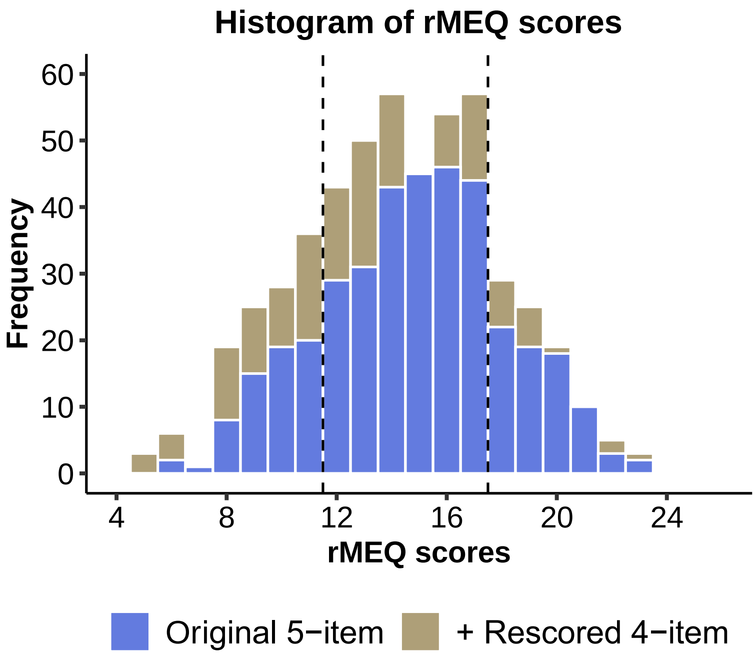

Figure S8. Frequency plot comparing the distribution of reduced Morningness-Eveningness Questionnaire (rMEQ) scores of individuals with the original 5-item rMEQ and those with the rescored 4-item rMEQ (n = 138). The scores of the subset of individuals with the 4-item rMEQ underwent rescaling: their 4-item rMEQ score was normalized (rMEQ 4-item score - 3) / (21 - 3)), scaled to the range of the 5-item version (* 25 – 4), and aligned with the 5-item version (+ 4). Dotted lines indicate the cut-off scores for the chronotype categories (11 or lower = evening-type, 18 or higher = morning-type, and scores between 12 and 17 = intermediate chronotypes).

SUPPLEMENTARY TABLES

Table S1. Number of completions for respective timepoint.

Time bin	N
08:00-<9:30	375
9:30-<12:00	348
12:00-<15:00	383
15:00-<18:30	379
18:30-<22:00	381
22:00 and later	366

Note. Please note all data were time stamped and the time stamps were used for the analyses. A number of people completed the timepoint later than the instructed time and therefore completed two timepoints within the shown time bin. The sample size of unique participant IDs per time bin (% of n = 410): 375 (91.5%), 340 (82.9%), 375 (91.5%), 374 (91.2%), 377 (92.0%), 366 (89.3%), respectively.

Table S2. Psychiatric symptoms and behaviors items, completed during the baseline session (to obtain the latent constructs) and the diurnal timepoints. Items were rated from 1 = Not at all" to "9 = Very much/All the time.

Baseline session: "in the past 3 hours:"
1st diurnal timepoint: "this morning:"
2nd diurnal timepoint: "since the last session:"
All other timepoints: "in the past 3 hours:"

1. Have you been interested or felt pleasure in doing things?
2. Have you felt down?
3. Have you felt irritated or angry?
4. Have you felt full of energy?
5. Have you felt anxious or nervous?
6. Have you had imposing unpleasant thoughts or images?
7. Have you had racing thoughts?
8. Have you had difficulties concentrating, or forgotten something?
9. Have you found it difficult to sit still?
10. Have you said or done things impulsively?
11. Have your emotions changed quickly, frequently, or a lot?
12. Have you had difficulties to get things done?
13. Have you felt stressed?
14. Have you had difficulties staying awake?
15. Have you been physically active?
16. Have you spent time with other people?
17. Have you spent time outside?

These questions regard how you feel right now:

18. How much would you like to rest?
19. How much would you like to be by yourself?
20. How much would you like to be with a group of friends?
21. How much would you like to work out/exercise?
22. How sleepy are you?

Table S3. Mean (*SD*) score and range for each psychiatric trait scale, showing the percentage of individuals potentially having clinical symptom levels.

	M (*SD*)	Observed range	Scale range	% above clinical cut-off / Indicative of clinical level
Depression	12.2 (6.6)	0-30	0-30	60.0%
Generalized anxiety	7.7 (5.2)	0-21	0-21	34.4%
Mania	4.5 (3.6)	0-18	0-20	37.6%
Delusional ideation	8.8 (6.0)	0-27	0-33	N/A
Emotion regulation difficulties	37.9 (15.0)	16-79	16-80	N/A
Autism	3.0 (2.1)	0-10	0-10	14.0%
Impulsivity	2.4 (0.7)	1-4	1-4	N/A
Emotional instability	19.7 (11.4)	0-48	0-54	N/A
ADHD (part A)	2.4 (1.7)	0-6	0-6	34.9%
OCD	17.5 (12.6)	0-59	0-72	33.5%
Eating disorder	10.6 (11.0)	0-56	0-78	19.7%
Apathy	36.3 (9.3)	18-63	18-72	42.1%
Social anxiety	57.2 (31.0)	0-144	0-144	54.8%

Note. Cut-off scores per scale: Depression ≥ 10; Generalized anxiety ≥ 10 (= moderate anxiety); Mania ≥ 6; Autism ≥ 6; ADHD (Attention Deficit Hyperactivity Disorder) ≥ 4 (based on Part A, 6 items); OCD (Obsessive Compulsive Disorder) ≥ 21; Eating disorder ≥ 20; Apathy ≥ 38; Social anxiety ≥ 50 (= moderate social anxiety); Delusional ideation consists of the PDI-21 Y/N items (*M* = 5.5, *SD* = 3.7, range = 0-17) and O-LIFE unusual experiences subscale. N/A = no cut-off value available or applicable. The sample consisted of a relatively high number of individuals with psychiatric trait levels above the questionnaire's indicative cut-off for a clinical disorder. This is potentially due to the timing of data collection during the COVID-19 pandemic (Oct 2021) when psychological well-being decreased in the general population [21]. Nevertheless, other online studies carried out prior to the COVID-19 pandemic found comparable scale distributions [22].

Table S4. Factor loadings of the psychiatric trait scales.

Psychiatric traits	Depression-Anxiety	Downregulatory problems	Compulsive behavior and intrusive thought	Social dysfunction
Depression	1.01	0.02	-0.03	-0.02
Generalized anxiety	0.74	0.01	0.17	0.05
Emotion regulation difficulties	0.36	0.19	0.21	0.31
ADHD	0.09	0.74	0.01	0.11
Impulsivity	-0.07	0.7	0.01	-0.14
Delusional ideation	0.11	0.09	0.62	-0.01
OCD	0.05	0.06	0.48	0.34
Mania	-0.37	0.07	0.37	-0.14
Emotional instability	0.29	0.32	0.34	0.07
Social anxiety	0.05	0.01	0.04	0.68
Autism	-0.13	0.31	-0.06	0.36
Apathy	0.33	0.12	-0.23	0.36
Eating disorder	0.01	0.02	0.27	0.29

Note. Higher factor loadings indicate stronger loadings for the respective construct, with both positive and negative loadings. Psychiatric trait scales are ordered according to strength of the factor loading.

Table S5. Transdiagnostic psychiatric trait construct correlations.

Psychiatric trait construct	Downregulatory problems	Social dysfunction	Compulsive behavior and intrusive thought
Depression-Anxiety	0.53	0.66	0.31
Downregulatory problems		0.46	0.47
Social dysfunction			0.28

Table S6. Symptom construct correlations.

Symptom construct	ADHD-type symptoms	Fatigue-related symptoms	Low drive and motivation
Negative emotional symptoms	0.70	0.38	0.17
ADHD-type symptoms		0.31	-0.03
Fatigue-related symptoms			0.19

 To table of contents

Table S7. Standardized coefficients and 95% confidence intervals (95% CI) for the relationships of reduced Morningness-Eveningness Questionnaire (rMEQ) score with psychiatric traits and transdiagnostic psychiatric trait constructs.

**Psychiatric traits**	Standardized coefficients (95% CI)	
	Univariable models	Multivariable model
Depression	-0.98*** [-1.28, -0.69]	-0.85** [-1.49, -0.21]
Generalized anxiety	-0.65*** [-0.95, -0.35]	0.66* [0.11, 1.21]
Mania	0.46** [0.16, 0.77]	0.20 [-0.13, 0.53]
Delusional ideation	-0.63*** [-0.94, -0.33]	-0.06# [-0.12, 0.00]
Emotion regulation difficulties	-0.83*** [-1.13, -0.53]	-0.03 [-0.54, 0.48]
Autism	-0.60*** [-0.91, -0.30]	-0.24 [-0.57, 0.09]
Impulsivity	-0.38* [-0.69, -0.07]	-0.02 [-0.37, 0.32]
Emotional instability	-0.84*** [-1.14, -0.53]	-0.31 [-0.77, 0.14]
ADHD	-0.78*** [-1.08, -0.48]	-0.10 [-0.54, 0.34]
OCD	-0.62*** [-0.93, -0.32]	-0.02 [-0.42, 0.38]
Eating disorder	-0.17 [-0.48, 0.14]	0.22 [-0.11, 0.55]
Apathy	-0.85*** [-1.15, -0.55]	-0.31 [-0.69, 0.06]
Social anxiety	-0.63*** [-0.94, -0.33]	-0.09 [-0.47, 0.28]
**Transdiagnostic psychiatric trait constructs**		
Depression-Anxiety	-0.98*** [-1.28, -0.69]	-0.53* [-0.93, -0.06]
Downregulatory problems	-0.87*** [-1.17, -0.57]	-0.43 [-0.88, 0.02]
Compulsive behavior and intrusive thought	-0.48** [-0.78, -0.17]	0.11 [-0.27, 0.50]
Social dysfunction	-0.94*** [-1.24, -0.64]	-0.32 [-0.80, 0.17]

Note. A lower rMEQ score indicates a stronger evening-type. Two multivariable models were specified: one including the 13 psychiatric traits and one including the four transdiagnostic psychiatric trait constructs. *** p <.001; ** p <.01; * p <.05, # p <.10

 To table of contents

Table S8. Relationships of chronotype with psychiatric traits and transdiagnostic psychiatric trait constructs (z-transformed). Results show odds ratios (OR) with 95% confidence intervals (CI) for evening-type and intermediate-type (morning-type served as reference category).

**Psychiatric trait**	OR (95% CI)	
	Evening-type	Intermediate-type
Depression	2.02*** [1.50, 2.71]	1.21 [0.94, 1.55]
Generalized anxiety	1.56** [1.18, 2.07]	1.08 [0.84, 1.38]
Mania	0.76# [0.58, 1.01]	0.92 [0.73, 1.15]
Delusional ideation	1.68*** [1.26, 2.24]	1.23 [0.95, 1.59]
Emotion regulation difficulties	1.80*** [1.34, 2.41]	1.27# [0.98, 1.65]
Autism	1.62*** [1.22, 2.15]	1.09 [0.85, 1.41]
Impulsivity	1.34* [1.02, 1.77]	1.07 [0.85, 1.36]
Emotional instability	1.86*** [1.40, 2.49]	1.36* [1.07, 1.73]
ADHD	1.90*** [1.42, 2.54]	1.28* [1.00, 1.64]
OCD	1.41* [1.07, 1.86]	1.06 [0.83, 1.36]
Eating disorder	1.20 [0.91, 1.58]	1.06 [0.83, 1.35]
Apathy	1.84*** [1.37, 2.46]	1.44** [1.11, 1.86]
Social anxiety	1.43* [1.08, 1.89]	1.12 [0.88, 1.43]
**Transdiagnostic psychiatric trait constructs**		
Depression-Anxiety	2.02*** [1.51, 2.71]	1.21 [0.94, 1.56]
Downregulatory problems	2.02*** [1.50, 2.71]	1.32* [1.02, 1.69]
Compulsive behavior and intrusive thought	1.44* [1.09, 1.91]	1.14 [0.89, 1.46]
Social dysfunction	1.87*** [1.39, 2.50]	1.26# [0.98, 1.63]

*** p <.001; ** p <.01; * p <.05; # p <.10.

Table S9. Relationships of chronotype and psychiatric traits and the transdiagnostic psychiatric trait constructs (z-transformed). Results show odds ratios (OR) corrected for age and gender with 95% confidence intervals (CI) for evening-type and intermediate-type (morning-type served as reference category).

**Psychiatric trait**	OR (95% CI)	
	Evening-type	Intermediate-type
Depression	2.02*** [1.50, 2.71]	1.20 [0.93, 1.54]
Age	0.74* [0.56, 0.97]	0.79* [0.64, 0.99]
Gender [Man]	1.47 [0.72, 2.99]	1.24 [0.68, 2.27]
Generalized anxiety	1.52** [1.14, 2.03]	1.05 [0.82, 1.35]
Age	0.76* [0.57, 1.00]	0.79* [0.63, 0.99]
Gender [Man]	1.39 [0.69, 2.81]	1.20 [0.66, 2.20]
Mania	0.70* [0.53, 0.93]	0.87 [0.69, 1.09]
Age	0.66** [0.50, 0.88]	0.76* [0.61, 0.96]
Gender [Man]	1.17 [0.58, 2.33]	1.17 [0.64, 2.12]
Delusional ideation	1.61** [1.20, 2.16]	1.20 [0.92, 1.55]
Age	0.77# [0.58, 1.02]	0.80# [0.64, 1.00]
Gender [Man]	1.23 [0.61, 2.47]	1.22 [0.67, 2.21]
Emotion regulation difficulties	1.74*** [1.29, 2.34]	1.23 [0.94, 1.60]
Age	0.79 [0.60, 1.05]	0.81# [0.60, 1.05]
Gender [Man]	1.40 [0.69, 2.83]	1.25 [0.68, 2.73]
Autism	1.53** [1.15, 2.04]	1.02 [0.79, 1.32]
Age	0.77# [0.58, 1.02]	0.79* [0.63, 0.99]
Gender [Man]	1.18 [0.52, 2.12]	1.18 [0.65, 2.14]
Impulsivity	1.30# [0.99, 1.72]	1.05 [0.82, 1.33]
Age	0.73* [0.56, 0.96]	0.79* [0.63, 0.98]
Gender [Man]	1.22 [0.61, 2.43]	1.19 [0.65, 2.16]
Emotional instability	1.83*** [1.35, 2.57]	1.32* [1.03, 1.71]
Age	0.82 [0.62, 1.09]	0.84 [0.66, 1.05]
Gender [Man]	1.58 [0.77, 3.22]	1.34 [0.73, 2.47]
ADHD	1.84*** [1.37, 2.47]	1.24# [0.96, 1.59]
Age	0.77# [0.58, 1.01]	0.80# [0.64, 1.01]
Gender [Man]	1.29 [0.64, 2.61]	1.22 [0.67, 2.22]
OCD	1.33# [1.00, 1.76]	1.01 [0.78, 1.30]
Age	0.76# [0.57, 1.00]	0.79* [0.63, 0.99]
Gender [Male]	1.24 [0.62, 2.48]	1.18 [0.65, 2.15]
Eating disorder	1.15 [0.87, 1.53]	1.02 [0.80, 1.32]
Age	0.73* [0.55, 0.96]	0.79* [0.63, 0.99]
Gender [Man]	1.27 [0.63, 2.55]	1.19 [0.65, 2.18]
Apathy	1.83*** [1.36, 2.46]	1.44** [1.11, 1.87]
Age	0.71* [0.54, 0.94]	0.78* [0.63, 0.98]

Table S9. Continued.

**Psychiatric trait**	OR (95% CI)	
	Evening-type	Intermediate-type
Gender [Man]	1.08 [0.54, 2.17]	1.11 [0.61, 2.03]
Social anxiety	1.39* [1.04, 1.85]	1.10 [0.86, 1.41]
Age	0.75* [0.57, 0.98]	0.80* [0.64, 1.00]
Gender [Man]	1.36 [0.67, 2.74]	1.23 [0.67, 2.25]
**Transdiagnostic psychiatric trait constructs**		
Depression-Anxiety	2.01*** [1.50, 2.71]	1.20 [0.93, 1.55]
Age	0.74* [0.56, 0.98]	0.80* [0.64, 0.99]
Gender [Man]	1.54 [0.75, 3.47]	1.24 [0.68, 2.27]
Downregulatory problems	1.94*** [1.44, 2.63]	1.26# [0.97, 1.62]
Age	0.81 [0.61, 1.07]	0.81# [0.65, 1.02]
Gender [Man]	1.35 [0.67, 2.74]	1.24 [0.68, 2.27]
Compulsive behavior and intrusive thought	1.35* [1.00, 1.82]	1.07 [0.83, 1.39]
Age	0.78# [0.59, 1.04]	0.80* [0.63, 1.01]
Gender [Man]	1.37 [0.65, 2.65]	1.21 [0.66, 2.22]
Social dysfunction	1.81*** [1.34, 2.43]	1.23 [0.94, 1.59]
Age	0.79# [0.60, 1.04]	0.81# [0.65, 1.02]
Gender [Man]	1.40 [0.69, 2.83]	1.24 [0.68, 2.27]

Note. Morning-types served as reference category for testing the chronotype effect, and woman served as reference category for testing the gender effect. For the gender analysis, two individuals who did not identify as either woman or man were excluded as two datapoints are deemed insufficient for a valid analysis. *** *p* <.001; ** *p* <.01; * *p* <.05; # *p* <.10.

Table S10. Factor loadings of the psychiatric symptoms and behaviors.

Psychiatric symptoms and behaviors	Negative emotional symptoms[1]	Fatigue-related symptoms[2]	Low drive and motivation[3]	ADHD-type symptoms[4]
Felt down[1]	**0.88**	0.02	-0.04	-0.05
Felt nervous or anxious[1]	**0.82**	-0.07	-0.03	0.04
Felt irritated or angry[1]	**0.74**	0.08	0.10	0.03
Felt stressed[1]	**0.74**	0.11	0.00	0.07
Had unpleasant thoughts[1]	**0.58**	-0.08	0.03	0.26
Sleepy[2]	-0.07	**0.84**	0.00	0.09
Would like to rest[2]	0.07	**0.80**	0.04	-0.06
Had difficulties staying awake[2]	0.02	**0.48**	-0.01	0.35
Have been physically active[3]	0.10	-0.01	**0.75**	-0.02
Spent time outside[3]	0.03	0.10	**0.69**	0.04
Spent time with others[3]	0.00	0.19	**0.51**	-0.10
Felt full of energy[3]	-0.20	-0.39	**0.47**	0.12
Been interested or felt pleasure[3]	-0.23	-0.11	**0.43**	0.12
Had difficulties to sit still[4]	-0.06	0.06	-0.01	**0.71**
Said or did things impulsively[4]	0.11	0.07	0.12	**0.66**
Had difficulties concentrating[4]	0.25	0.16	-0.11	**0.49**
Had emotions changing[4]	0.42	0.03	0.05	**0.49**
Had racing thoughts[4]	0.41	-0.11	-0.02	**0.48**
Had difficulties getting things done[4]	0.25	0.19	-0.28	**0.42**
Would like to work out or exercise	-0.09	-0.10	0.18	0.13
Would like to be by myself	0.20	0.26	-0.04	0.01

Note. Higher factor loadings indicate stronger loadings for the respective construct, with both positive and negative loadings. Psychiatric symptoms are ordered according to strength of the factor loading. The numbers represent the primary factors to which the symptoms load the strongest, also indicated with bold text.

Table S11. GAMM results for predicting changes in negative emotional symptoms across the day from chronotype.

Negative emotional symptoms				
Parametric coefficients	Estimate	SE	T value	*p*-value
Intercept	1.27	0.15	8.62	<2e-16***
Intermediate-type	0.25	0.17	1.50	0.135
Evening-type	0.54	0.20	2.	0.007**
Smooth terms	EDF	RefDF	F-value	*p*-value
ID	3.67e+02	408	9.08	<2e-16***
Time-of-day	2.84e+00	4	15.29	5.60e-05***
Time-of-day x morning-type	9.49e-01	4	5.88	0.000***
Time-of-day x intermediate-type	9.53e-01	4	5.12	3.08e-05***
Time-of-day x evening-type	5.38e-05	4	0.00	0.997

 To table of contents

Table S12. GAMM results for predicting changes in fatigue-related symptoms across the day from chronotype.

Fatigue-related symptoms				
Parametric coefficients	Estimate	SE	T value	*p*-value
Intercept	3.17	0.15	21.03	<2e-16***
Intermediate-type	0.26	0.17	1.50	0.133
Evening-type	0.30	0.20	1.45	0.148
Smooth terms	EDF	RefDF	F-value	*p*-value
ID	3.19e+02	408	3.71	<2e-16***
Time-of-day	3.85e+00	4	163.11	<2e-16***
Time-of-day x morning-type	1.70e+00	4	10.91	0.000***
Time-of-day x intermediate-type	2.82e-03	4	0.00	0.928
Time-of-day x evening-type	1.26e+00	4	10.75	<2e-16***

Table S13. GAMM results for predicting changes in low drive and motivation across the day from chronotype.

Low drive and motivation				
Parametric coefficients	Estimate	SE	T value	*p*-value
Intercept	3.55	0.12	30.55	<2e-16***
Intermediate-type	-0.30	0.13	-2.30	0.022*
Evening-type	-0.82	0.16	-5.21	2.06e-07***
Smooth terms	EDF	RefDF	F-value	*p*-value
ID	307.65	408	3.08	<2e-16***
Time-of-day	2.68	4	37.62	<2e-16***
Time-of-day x morning-type	0.91	4	17.22	9.91e-07***
Time-of-day x intermediate-type	1.68	4	1.48	0.049*
Time-of-day x evening-type	1.217	4	0.87	0.063

Table S14. GAMM results for predicting changes in ADHD-type symptoms across the day from chronotype.

ADHD-type symptoms				
Parametric coefficients	Estimate	SE	T value	*p*-value
Intercept	1.13	0.12	9.20	<2e-16***
Intermediate-type	0.16	0.14	1.16	0.247
Evening-type	0.31	0.17	1.84	0.065
Smooth terms	EDF	RefDF	F-value	*p*-value
ID	3.68e+02	408	9.40	<2e-16***
Time-of-day	2.65e+00	4	8.92	0.001***
Time-of-day x morning-type	1.01e+00	4	0.81	0.188
Time-of-day x intermediate-type	4.93e-05	4	0.00	0.983
Time-of-day x evening-type	9.43e-01	4	5.68	0.004**

 To table of contents

Model comparisons

Table S15. GAMM comparisons for predicting changes in negative emotional symptoms across the day from depression-anxiety (D-A) construct and chronotype.

Depression-Anxiety (D-A) construct<br>Negative emotional Symptoms				
New predictor over previous model	AIC	Edf	fREML	*p*-value
ID	2367	NA	NA	NA
Time-of-day	2263	4	1448	<2e-16
D-A construct	2238	6	1333	<2e-16
Chronotype	2238	6	1333	NA
D-A x Time-of-day	2240	11	1336	0.982
Time-of-day x Chronotype	2239	17	1335	0.967
D-A construct x Chronotype	2239	23	1334	0.728
**D-A construct x Time-of-day x Chronotype**	**2230**	**32**	**1328**	**0.212**

Note. GAMM = generalized additive mixed-effect model, AIC = Akaike information criterion, Edf = estimated degrees of freedom, fREML = fast restricted maximum likelihood score. Bold row represents the model with the best fit (based on AIC).

Table S16. GAMM comparisons for predicting changes in fatigue-related symptoms across the day from depression-anxiety (D-A) construct and chronotype.

Depression-Anxiety (D-A) construct<br>Fatigue-related symptoms				
New predictor over previous model	AIC	Edf	fREML	*p*-value
ID	9974	NA	NA	NA
Time-of-day	9312	4	4818	<2e-16
D-A construct	9301	6	4793	1.037e-11
Chronotype	9301	6	4793	NA
D-A construct x Time-of-day	9261	11	4780	5.060e-06
Time-of-day x Chronotype	9163	17	4737	2.326e-16
D-A construct x Chronotype	9165	23	4737	0.999
**D-A construct x Time-of-day x Chronotype**	**9123**	**32**	**4721**	**1.712e-04**

Note. GAMM = generalized additive mixed-effect model, AIC = Akaike information criterion, Edf = estimated degrees of freedom, fREML = fast restricted maximum likelihood score. Bold row represents the model with the best fit (based on AIC).

 To table of contents

Table S17. GAMM comparisons for predicting changes in low drive and motivation across the day from depression-anxiety (D-A) construct and chronotype.

Depression-Anxiety (D-A) construct<br>Low drive and motivation				
New predictor over previous model	AIC	Edf	fREML	*p*-value
ID	8524	NA	NA	NA
Time-of-day	8279	4	4297	<2e-16
D-A construct	8269	6	4274	1.752e-10
Chronotype	8266	8	4267	6.221e-04
D-A construct x Time-of-day	8259	11	4264	0.118
Time-of-day x Chronotype	8231	17	4250	1.096e-04
D-A construct x Chronotype	8231	17	4250	NA
**D-A construct x Time-of-day x Chronotype**	**8224**	**32**	**4247**	**0.677**

Note. GAMM = generalized additive mixed-effect model, AIC = Akaike information criterion, Edf = estimated degrees of freedom, fREML = fast restricted maximum likelihood score. Bold row represents the model with the best fit (based on AIC).

Table S18. GAMM comparisons for predicting changes in ADHD-type symptoms across the day from depression-anxiety (D-A) construct and chronotype.

Depression-Anxiety (D-A) construct<br>ADHD-type symptoms				
New predictor over previous model	AIC	Edf	fREML	*p*-value
ID	1684	NA	NA	NA
Time-of-day	1651	4	1141	4.867e-06
D-A construct	1633	6	1052	<2e-16
Chronotype	1633	6	1052	NA
D-A construct x Time-of-day	1632	11	1054	0.525
**Time-of-day x Chronotype**	**1628**	**17**	**1051**	**0.360**
D-A construct x Chronotype	1628	23	1051	1.000
D-A construct x Time-of-day x Chronotype	1633	32	1046	0.522

Note. GAMM = generalized additive mixed-effect model, AIC = Akaike information criterion, Edf = estimated degrees of freedom, fREML = fast restricted maximum likelihood score. Bold row represents the model with the best fit (based on AIC).

Table S19. GAMM comparisons for predicting changes in negative emotional symptoms across the day from downregulatory problems (DP) construct and chronotype.

Downregulatory problems construct<br>Negative emotional symptoms				
New predictor over previous model	AIC	Edf	fREML	*p*-value
ID	2367	NA	NA	NA
Time-of-day	2263	4	1448	< 2e-16
DP construct	2255	6	1406	< 2e-16
Chronotype	2255	6	1406	NA
DP construct x Time-of-day	2252	11	1407	0.352
Time-of-day x Chronotype	2251	17	1407	0.945
DP construct x Chronotype	2251	17	1407	NA
**DP construct x Time-of-day x Chronotype**	**2247**	**32**	**1404**	**0.778**

Note. GAMM = generalized additive mixed-effect model, AIC = Akaike information criterion, Edf = estimated degrees of freedom, fREML = fast restricted maximum likelihood score. Bold row represents the model with the best fit (based on AIC).

To table of contents

Table S20. GAMM comparisons for predicting changes in fatigue-related symptoms across the day from downregulatory problems (DP) construct and chronotype.

Downregulatory problems (DP) construct<br>Fatigue-related symptoms				
New predictor over previous model	AIC	Edf	fREML	*p*-value
ID	9974	NA	NA	NA
Time-of-day	9312	4	4818	<2e-16
DP construct	9306	6	4805	1.168e-06
Chronotype	9307	6	4805	NA
DP construct x Time-of-day	9282	11	4796	3.161e-04
Time-of-day x Chronotype	9167	17	4748	<2e-16
DP construct x Chronotype	9167	23	4747	0.974
**DP construct x Time-of-day x Chronotype**	**9164**	**32**	**4746**	**0.964**

Note. GAMM = generalized additive mixed-effect model, AIC = Akaike information criterion, Edf = estimated degrees of freedom, fREML = fast restricted maximum likelihood score. Bold row represents the model with the best fit (based on AIC).

Table S21. GAMM comparisons for predicting changes in low drive and motivation across the day from downregulatory problems (DP) construct and chronotype.

Downregulatory problems construct<br>Low drive and motivation				
New predictor over previous model	AIC	Edf	fREML	*p*-value
ID	8524	NA	NA	NA
Time-of-day	8279	4	4297	<2e-16
DP construct	8275	6	4289	3.133e-04
Chronotype	8271	8	4279	7.328e-05
DP construct x Time-of-day	8267	11	4277	0.248
Time-of-day x Chronotype	8243	17	4264	1.378e-04
**DP construct x Chronotype**	**8240**	**23**	**4263**	**0.999**
DP construct x Time-of-day x Chronotype	8244	32	4262	0.946

Note. GAMM = generalized additive mixed-effect model, AIC = Akaike information criterion, Edf = estimated degrees of freedom, fREML = fast restricted maximum likelihood score. Bold row represents the model with the best fit (based on AIC).

Table S22. GAMM comparisons for predicting changes in ADHD-type symptoms across the day from downregulatory problems (DP) construct and chronotype.

Downregulatory problems (DP) construct<br>ADHD-type symptoms				
New predictor over previous model	AIC	Edf	fREML	*p*-value
ID	1684	NA	NA	NA
Time-of-day	1651	4	1141	4.867e-06
DP construct	1637	6	1067	<2e-16
Chronotype	1637	6	1067	NA
DP construct x Time-of-day	1627	11	1066	0.029
Time-of-day x Chronotype	1621	17	1063	0.421
DP construct x Chronotype	1622	23	1063	1.000
**DP construct x Time-of-day x Chronotype**	**1602**	**32**	**1056**	**0.096**

Note. GAMM = generalized additive mixed-effect model, AIC = Akaike information criterion, Edf = estimated degrees of freedom, fREML = fast restricted maximum likelihood score. Bold row represents the model with the best fit (based on AIC).

Table S23. GAMM comparisons for predicting changes in negative emotional symptoms across the day from compulsive behavior and intrusive thought (CBIT) construct and chronotype.

Compulsive behavior and intrusive thought (CBIT)<br>Negative emotional Symptoms				
New predictor over previous model	AIC	Edf	fREML	*p*-value
ID	2367	NA	NA	NA
Time-of-day	2263	4	1448	<2e-16
CBIT construct	2258	6	1421	2.625e-12
Chronotype	2258	6	1421	NA
CBIT construct x Time-of-day	2251	11	1421	0.175
**Time-of-day x Chronotype**	**2249**	**17**	**1420**	**0.929**
CBIT construct x Chronotype	2249	17	1420	NA
CBIT construct x Time-of-day x Chronotype	2257	32	1420	1.000

Note. GAMM = generalized additive mixed-effect model, AIC = Akaike information criterion, Edf = estimated degrees of freedom, fREML = fast restricted maximum likelihood score. Bold row represents the model with the best fit (based on AIC).

Table S24. GAMM comparisons for predicting changes in fatigue-related symptoms across the day from compulsive behavior and intrusive thought (CBIT) construct and chronotype.

Compulsive behavior and intrusive thought (CBIT) construct<br>Fatigue-related symptoms				
New predictor over previous model	AIC	Edf	fREML	*p*-value
ID	9974	NA	NA	NA
Time-of-day	9312	4	4818	<2e-16
CBIT construct	9310	6	4812	0.002
Chronotype	9310	6	4812	NA
CBIT construct x Time-of-day	9309	11	4812	0.589
**Time-of-day x Chronotype**	**9182**	**17**	**4759**	**<2e-16**
CBIT construct x Chronotype	9183	23	4758	0.700
CBIT construct x Time-of-day x Chronotype	9182	32	4757	1.000

Note. GAMM = generalized additive mixed-effect model, AIC = Akaike information criterion, Edf = estimated degrees of freedom, fREML = fast restricted maximum likelihood score. Bold row represents the model with the best fit (based on AIC).

Table S25. GAMM comparisons for predicting changes in low drive and motivation across the day from compulsive behavior and intrusive thought (CBIT) construct and chronotype.

Compulsive behavior and intrusive thought (CBIT) construct<br>Low drive and motivation				
New predictor over previous model	AIC	Edf	fREML	*p*-value
ID	8524	NA	NA	NA
Time-of-day	8279	4	4297	<2e-16
CBIT construct	8280	6	4297	0.931
Chronotype	8275	8	4284	3.289e-06
CBIT construct x Time-of-day	8275	11	4283	0.618
**Time-of-day x Chronotype**	**8235**	**17**	**4267**	**1.367e-05**
CBIT construct x Chronotype	8235	23	4266	0.956
CBIT construct x Time-of-day x Chronotype	8235	23	4266	NA

Note. GAMM = generalized additive mixed-effect model, AIC = Akaike information criterion, Edf = estimated degrees of freedom, fREML = fast restricted maximum likelihood score. Bold row represents the model with the best fit (based on AIC).

Table S26. GAMM comparisons for predicting changes in ADHD-type symptoms across the day from compulsive behavior and intrusive thought (CBIT) construct and chronotype.

Compulsive behavior and intrusive thought (CBIT) construct<br>ADHD-type symptoms				
New predictor over previous model	AIC	Edf	fREML	*p*-value
ID	1684	NA	NA	NA
Time-of-day	1651	4	1141	4.867e-06
CBIT construct	1644	6	1102	<2e-16
Chronotype	1644	6	1102	NA
CBIT construct x Time-of-day	1639	11	1103	0.311
Time-of-day x Chronotype	1636	17	1100	0.368
CBIT construct x Chronotype	1637	23	1100	1.000
**CBIT construct x Time-of-day x Chronotype**	**1632**	**32**	**1098**	**0.930**

Note. GAMM = generalized additive mixed-effect model, AIC = Akaike information criterion, Edf = estimated degrees of freedom, fREML = fast restricted maximum likelihood score. Bold row represents the model with the best fit (based on AIC).

Table S27. GAMM comparisons for predicting changes in negative emotional symptoms across the day from social dysfunction (SD) construct and chronotype.

Social Dysfunction (SD) construct<br>Negative emotional Symptoms				
New predictor over previous model	AIC	Edf	fREML	*p*-value
ID	2367	NA	NA	NA
Time-of-day	2263	4	1448	<2e-16
SD construct	2245	6	1359	<2e-16
Chronotype	2245	6	1359	NA
SD construct x Time-of-day	2236	11	1360	0.127
Time-of-day x Chronotype	2238	17	1360	0.984
SD construct x Chronotype	2238	23	1360	1.000
**SD construct x Time-of-day x Chronotype**	**2230**	**32**	**1356**	**0.685**

Note. GAMM = generalized additive mixed-effect model, AIC = Akaike information criterion, Edf = estimated degrees of freedom, fREML = fast restricted maximum likelihood score. Bold row represents the model with the best fit (based on AIC).

Table S28. GAMM comparisons for predicting changes in fatigue-related symptoms across the day from social dysfunction construct and chronotype.

Social Dysfunction (SD) construct<br>Fatigue-related symptoms				
New predictor over previous model	AIC	Edf	fREML	*p*-value
ID	9974	NA	NA	NA
Time-of-day	9312	4	4818	<2e-16
SD construct	9302	6	4796	1.479e-10
Chronotype	9303	6	4796	NA
SD construct x Time-of-day	9281	11	4788	8.202e-04
Time-of-day x Chronotype	9170	17	4741	<2e-16
SD construct x Chronotype	9170	23	4740	0.926
**SD construct x Time-of-day x Chronotype**	**9138**	**32**	**4730**	**0.011**

Note. GAMM = generalized additive mixed-effect model, AIC = Akaike information criterion, Edf = estimated degrees of freedom, fREML = fast restricted maximum likelihood score. Bold row represents the model with the best fit (based on AIC).

Table S29. GAMM comparisons for predicting changes in low drive and motivation across the day from social dysfunction (SD) construct and chronotype.

Social Dysfunction (SD) construct<br>Low drive and motivation				
New predictor over previous model	AIC	Edf	fREML	*p*-value
ID	8524	NA	NA	NA
Time-of-day	8279	4	4297	<2e-16
SD construct	8268	6	4273	4.631e-11
Chronotype	8265	8	4265	5.024e-04
SD construct x Time-of-day	8268	11	4264	0.407
Time-of-day x Chronotype	8234	17	4250	9.320e-05
**SD construct x Chronotype**	**8231**	**17**	**4250**	**NA**
SD construct x Time-of-day x Chronotype	8231	32	4248	0.895

Note. GAMM = generalized additive mixed-effect model, AIC = Akaike information criterion, Edf = estimated degrees of freedom, fREML = fast restricted maximum likelihood score. Bold row represents the model with the best fit (based on AIC).

Table S30. GAMM comparisons for predicting changes in ADHD-type symptoms across the day from social dysfunction (SD) construct and chronotype.

Social Dysfunction (SD) construct<br>ADHD-type symptoms				
New predictor over previous model	AIC	Edf	fREML	*p*-value
ID	1684	NA	NA	NA
Time-of-day	1651	4	1141	4.867e-06
SD construct	1633	6	1053	<2e-16
Chronotype	1634	6	1053	NA
SD construct x Time-of-day	1624	11	1052	0.072
Time-of-day x Chronotype	1627	17	1050	0.584
SD construct x Chronotype	1628	23	1050	1.000
**SD construct x Time-of-day x Chronotype**	**1623**	**32**	**1048**	**0.942**

Note. GAMM = generalized additive mixed-effect model, AIC = Akaike information criterion, Edf = estimated degrees of freedom, fREML = fast restricted maximum likelihood score. Bold row represents the model with the best fit (based on AIC).

Model results

Table S31. GAMM results of the associations of negative emotional symptoms with Depression-Anxiety (D-A), chronotype, and time-of-day.

Depression-Anxiety (D-A) construct – Negative emotional symptoms				
Parametric coefficients	Estimate	SE	T value	*p*-value
Intercept	0.75	0.04	18.05	<2e-16***
Intermediate-type	0.03	0.05	0.63	0.532
Evening-type	-0.04	0.06	-0.79	0.430
Smooth terms	EDF	RefDF	F-value	*p*-value
ID	335.40	407	4.93	<2e-16***
Time-of-day	3.77	4	28.91	<2e-16***
D-A construct	1.00	4	1822.28	<2e-16***
D-A construct x time-of-day	0.00	16	0.00	0.878
Time-of-day x morning-type	0.44	4	0.20	0.177
Time-of-day x intermediate-type	0.00	4	0.00	0.935
Time-of-day x evening-type	0.32	4	0.13	0.219
D-A construct x morning-type	0.00	4	0.00	0.806
D-A construct x intermediate-type	0.00	4	0.00	0.639
D-A construct x evening-type	0.84	4	35.90	0.120
D-A construct x time-of-day x morning-type	1.25	16	0.17	0.114
D-A construct x time-of-day x intermediate-type	0.92	16	0.09	0.193
D-A construct x time-of-day x evening-type	3.34	16	1.36	0.000***

Note. EDF = effective degrees of freedom; RefDF = reference degrees of freedom. Morning-type served as reference category for the parametric coefficients.

Table S32. GAMM results of the associations of fatigue-related symptoms with Depression-Anxiety (D-A) construct, chronotype, and time-of-day.

Depression-Anxiety (D-A) construct – Fatigue-related symptoms				
Parametric coefficients	Estimate	SE	T value	*p*-value
Intercept	3.32	0.14	23.54	<2e-16***
Intermediate-type	0.13	0.16	0.84	0.401
Evening-type	-0.18	0.19	-0.91	0.363
Smooth terms	EDF	RefDF	F-value	*p*-value
ID	303.50	407	3.05	<2e-16***
Time-of-day	3.73	4	149.84	<2e-16***
D-A construct	2.86	4	216.45	<2e-16***
D-A construct x time-of-day	0.93	16	0.12	0.079
Time-of-day x morning-type	2.00	4	32.26	<2e-16***
Time-of-day x intermediate-type	0.99	4	23.98	<2e-16***
Time-of-day x evening-type	0.00	4	0.00	0.426
D-A construct x morning-type	0.00	4	0.00	0.364
D-A construct x intermediate-type	0.00	4	0.00	0.535
D-A construct x evening-type	1.16	4	4.91	0.304
D-A construct x time-of-day x morning-type	2.18	12	1.43	0.000***
D-A construct x time-of-day x intermediate-type	1.08	16	0.12	0.107
D-A construct x time-of-day x evening-type	2.48	11	3.71	0.000***

Note. EDF = effective degrees of freedom; RefDF = reference degrees of freedom. Morning-type served as reference category for the parametric coefficients.

Table S33. GAMM results of the associations of low drive and motivation with Depression-Anxiety (D-A) construct, chronotype, and time-of-day.

Depression-Anxiety (D-A) construct – Low drive and motivation				
Parametric coefficients	Estimate	SE	T value	*p*-value
Intercept	3.44	0.11	30.57	<2e-16***
Intermediate-type	-0.19	0.13	-1.48	0.140
Evening-type	-0.60	0.15	-3.92	0.000***
Smooth terms	EDF	RefDF	F-value	*p*-value
ID	297.30	407	2.79	<2e-16***
Time-of-day	2.70	4	46.85	<2e-16***
D-A construct	0.98	4	137.43	<2e-16***
D-A construct x time-of-day	0.24	16	0.02	0.293
Time-of-day x morning-type	0.97	4	10.30	<2e-16***
Time-of-day x intermediate-type	0.91	4	2.87	0.000***
Time-of-day x evening-type	1.19	4	0.62	0.128
D-A construct x morning-type	0.00	4	0.00	0.971
D-A construct x intermediate-type	0.00	4	0.00	0.505
D-A construct x evening-type	0.00	4	0.00	0.630
D-A construct x time-of-day x morning-type	2.73	16	0.64	0.006**
D-A construct x time-of-day x intermediate-type	0.00	16	0.00	0.699
D-A construct x time-of-day x evening-type	2.62	16	1.01	0.005**

Note. EDF = effective degrees of freedom; RefDF = reference degrees of freedom. Morning-type served as reference category for the parametric coefficients.

Table S34. GAMM results of the associations of ADHD-type symptoms with Depression-Anxiety (D-A) construct, chronotype, and time-of-day.

Depression-Anxiety (D-A) construct – ADHD-type symptoms				
Parametric coefficients	Estimate	SE	T value	*p*-value
Intercept	0.69	0.04	17.61	<2e-16***
Intermediate-type	0.03	0.04	0.63	0.530
Evening-type	-0.03	0.05	-0.65	0.518
Smooth terms	EDF	RefDF	F-value	*p*-value
ID	345.90	407	5.95	<2e-16***
Time-of-day	2.53	4	8.06	0.000***
D-A construct	1.00	4	2554.62	0.000***
D-A construct x time-of-day	2.51	16	0.32	0.107
Time-of-day x morning-type	0.47	4	0.20	0.189
Time-of-day x intermediate-type	0.00	4	0.00	0.970
Time-of-day x evening-type	0.89	4	2.95	0.007**

Note. EDF = effective degrees of freedom; RefDF = reference degrees of freedom. Morning-type served as reference category for the parametric coefficients.

Table S35. GAMM results of the associations of negative emotional symptoms with Downregulatory Problems (DP) construct, chronotype, and time-of-day.

Downregulatory Problems (DP) construct – Negative emotional symptoms				
Parametric coefficients	Estimate	SE	T value	*p*-value
Intercept	0.71	0.05	14.34	<2e-16***
Intermediate-type	0.06	0.06	1.10	0.272
Evening-type	0.06	0.07	0.85	0.394
Smooth terms	EDF	RefDF	F-value	*p*-value
ID	356.00	407	7.40	<2e-16***
Time-of-day	3.77	4	31.99	<2e-16***
DP construct	2.68	4	1590.72	<2e-16***
DP construct x time-of-day	0.00	16	0.00	0.422
Time-of-day x morning-type	0.19	4	0.06	0.263
Time-of-day x intermediate-type	0.00	4	0.00	0.865
Time-of-day x evening-type	0.60	4	0.45	0.112
DP construct x morning-type	0.00	4	0.00	0.457
DP construct x intermediate-type	0.00	4	0.00	0.965
DP construct x evening-type	0.00	4	0.00	0.989
DP construct x time-of-day x morning-type	0.00	16	0.00	0.660
DP construct x time-of-day x intermediate-type	1.45	16	0.59	0.007
DP construct x time-of-day x evening-type	3.27	16	0.84	0.032*

Note. EDF = effective degrees of freedom; RefDF = reference degrees of freedom. Morning-type served as reference category for the parametric coefficients.

Table S36. GAMM results of the associations of fatigue-related symptoms with Downregulatory Problems (DP) construct, chronotype, and time-of-day.

Downregulatory Problems (DP) construct – Fatigue-related symptoms				
Parametric coefficients	Estimate	SE	T value	*p*-value
Intercept	3.25	0.15	22.32	<2e-16***
Intermediate-type	0.19	0.17	1.16	0.247
Evening-type	0.04	0.20	0.18	0.856
Smooth terms	EDF	RefDF	F-value	*p*-value
ID	310.50	407	3.39	<2e-16***
Time-of-day	3.85	4	180.00	<2e-16***
DP construct	0.92	4	41.93	0.001***
DP construct x time-of-day	1.53	16	0.54	0.004**
Time-of-day x morning-type	1.79	4	7.04	0.000***
Time-of-day x intermediate-type	0.00	4	0.00	0.974
Time-of-day x evening-type	0.98	4	16.13	<2e-16***
DP construct x morning-type	0.00	4	0.00	0.917
DP construct x intermediate-type	1.32	4	18.11	0.017*
DP construct x evening-type	0.00	4	0.00	0.813
DP construct x time-of-day x morning-type	0.81	15	0.32	0.025*
DP construct x time-of-day x intermediate-type	0.00	16	0.00	0.516
DP construct x time-of-day x evening-type	0.49	16	0.07	0.185

Note. EDF = effective degrees of freedom; RefDF = reference degrees of freedom. Morning-type served as reference category for the parametric coefficients.

Table S37. GAMM results of the associations of low drive and motivation with Downregulatory Problems (DP) construct, chronotype, and time-of-day.

Downregulatory Problems (DP) construct – Low drive and motivation				
Parametric coefficients	Estimate	SE	T value	*p*-value
Intercept	3.50	0.12	30.11	<2e-16***
Intermediate-type	-0.24	0.13	-1.83	0.068
Evening-type	-0.72	0.16	-4.58	0.000***
Smooth terms	EDF	RefDF	F-value	*p*-value
ID	303.00	407	3.14	<2e-16***
Time-of-day	2.70	4	41.57	<2e-16***
DP construct	0.79	4	9.09	0.262
DP construct x time-of-day	1.40	16	0.25	0.053
Time-of-day x morning-type	1.47	4	17.20	<2e-16***
Time-of-day x intermediate-type	0.91	4	2.88	0.001***
Time-of-day x evening-type	1.27	4	0.69	0.121
DP construct x morning-type	0.00	4	0.00	0.959
DP construct x intermediate-type	0.59	4	2.93	0.413
DP construct x evening-type	0.00	4	0.00	0.942

Note. EDF = effective degrees of freedom; RefDF = reference degrees of freedom. Morning-type served as reference category for the parametric coefficients.

Table S38. GAMM results of the associations of ADHD-type symptoms with Downregulatory Problems (DP) construct, chronotype, and time-of-day.

Downregulatory Problems (DP) construct – ADHD-type symptoms				
Parametric coefficients	Estimate	SE	T value	*p*-value
Intercept	0.67	0.04	16.54	<2e-16***
Intermediate-type	0.05	0.05	1.03	0.302
Evening-type	-0.01	0.05	-0.15	0.881
Smooth terms	EDF	RefDF	F-value	*p*-value
ID	349.20	407	6.37	<2e-16***
Time-of-day	2.53	4	8.28	0.000***
DP construct	2.14	4	1548.65	<2e-16***
DP construct x time-of-day	1.40	16	0.35	0.027*
Time-of-day x morning-type	0.61	4	0.41	0.108
Time-of-day x intermediate-type	0.00	4	0.00	0.965
Time-of-day x evening-type	0.74	4	0.86	0.047*
DP construct x morning-type	0.12	4	0.25	0.342
DP construct x intermediate-type	0.00	4	0.00	0.827
DP construct x evening-type	0.40	4	1.63	0.625
DP construct x time-of-day x morning-type	0.87	16	0.17	0.155
DP construct x time-of-day x intermediate-type	0.89	15	0.17	0.080
DP construct x time-of-day x evening-type	0.94	16	1.31	0.000***

Note. EDF = effective degrees of freedom; RefDF = reference degrees of freedom. Morning-type served as reference category for the parametric coefficients.

Table S39. GAMM results of the associations of negative emotional symptoms with Compulsive Behavior and Intrusive Thought (CBIT) construct, chronotype, and time-of-day.

Compulsive Behavior and Intrusive Thought (CBIT) construct – Negative emotional symptoms				
Parametric coefficients	Estimate	SE	T value	*p*-value
Intercept	0.68	0.05	13.23	<2e-16***
Intermediate-type	0.09	0.06	1.49	0.137
Evening-type	0.13	0.07	1.88	0.061
Smooth terms	EDF	RefDF	F-value	*p*-value
ID	359.90	407	8.58	<2e-16***
Time-of-day	3.77	4	33.32	<2e-16***
CBIT construct	2.03	4	1180.88	0.341
CBIT construct x time-of-day	1.63	10	0.92	0.009**
Time-of-day x morning-type	0.00	4	0.00	0.487
Time-of-day x intermediate-type	0.00	4	0.00	0.845
Time-of-day x morning-type	0.77	4	1.07	0.038*

Note. EDF = effective degrees of freedom; RefDF = reference degrees of freedom. Morning-type served as reference category for the parametric coefficients.

Table S40. GAMM results of the associations of fatigue-related symptoms with Compulsive Behavior and Intrusive Thought (CBIT) construct, chronotype, and time-of-day.

Compulsive Behavior and Intrusive Thought (CBIT) construct – Fatigue-related symptoms				
Parametric coefficients	Estimate	SE	T value	*p*-value
Intercept	3.22	0.15	21.69	<2e-16***
Intermediate-type	0.22	0.17	1.31	0.190
Evening-type	0.12	0.20	0.61	0.542
Smooth terms	EDF	RefDF	F-value	*p*-value
ID	314.50	407	3.56	<2e-16***
Time-of-day	3.85	4	180.27	<2e-16***
CBIT construct	1.37	4	64.43	0.129
CBIT construct x time-of-day	2.26	16	0.30	0.083
Time-of-day x morning-type	1.82	4	9.15	<2e-16***
Time-of-day x intermediate-type	0.00	4	0.00	0.994
Time-of-day x evening-type	0.98	4	20.22	<2e-16***

Note. EDF = effective degrees of freedom; RefDF = reference degrees of freedom. Morning-type served as reference category for the parametric coefficients.

Table S41. GAMM results of the associations of low drive and motivation with Compulsive Behavior and Intrusive Thought (CBIT) construct, chronotype, and time-of-day.

Compulsive Behavior and Intrusive Thought (CBIT) construct – Low drive and motivation				
Parametric coefficients	Estimate	SE	T value	*p*-value
Intercept	3.52	0.12	30.04	<2e-16***
Intermediate-type	-0.25	0.13	-1.91	0.057
Evening-type	-0.78	0.16	-5.00	0.000***
Smooth terms	EDF	RefDF	F-value	*p*-value
ID	307.44	407	3.19	<2e-16***
Time-of-day	2.71	4	44.16	<2e-16***
CBIT construct	0.15	4	0.11	0.673
CBIT construct x time-of-day	3.58	16	0.81	0.008**
Time-of-day x morning-type	1.41	4	18.77	<2e-16***
Time-of-day x intermediate-type	0.91	4	2.87	0.001***
Time-of-day x evening-type	1.04	4	0.52	0.143

Note. EDF = effective degrees of freedom; RefDF = reference degrees of freedom. Morning-type served as reference category for the parametric coefficients.

Table S42. GAMM results of the associations of ADHD-type symptoms with Compulsive Behavior and Intrusive Thought (CBIT) construct, chronotype, and time-of-day.

Compulsive Behavior and Intrusive Thought (CBIT) construct – ADHD-type symptoms				
Parametric coefficients	Estimate	SE	T value	*p*-value
Intercept	0.63	0.04	14.35	<2e-16***
Intermediate-type	0.08	0.05	1.57	0.116
Evening-type	0.09	0.06	1.45	0.147
Smooth terms	EDF	RefDF	F-value	*p*-value
ID	358.20	407	7.87	<2e-16***
Time-of-day	2.55	4	8.51	0.000***
SD construct	1.77	4	1375.97	<2e-16***
SD construct x time-of-day	0.00	16	0.00	0.415
Time-of-day x morning-type	0.64	4	0.30	0.164
Time-of-day x intermediate-type	0.00	4	0.00	0.972
Time-of-day x evening-type	0.89	4	2.56	0.002**
CBIT construct x morning-type	0.14	4	0.49	0.774
CBIT construct x intermediate-type	0.00	4	0.00	0.702
CBIT construct x evening-type	0.00	4	0.00	0.886
CBIT construct x time-of-day x morning-type	0.99	9	0.29	0.122
CBIT construct x time-of-day x intermediate-type	0.96	14	0.20	0.109
CBIT construct x time-of-day x evening-type	1.41	9	0.89	0.011*

Note. EDF = effective degrees of freedom; RefDF = reference degrees of freedom. Morning-type served as reference category for the parametric coefficients.

Table S43. GAMM results of the associations of negative emotional symptoms with Social Dysfunction (SD) construct, chronotype, and time-of-day.

Social Dysfunction (SD) construct – Negative emotional symptoms				
Parametric coefficients	Estimate	SE	T value	*p*-value
Intercept	0.73	0.04	16.49	<2e-16***
Intermediate-type	0.05	0.05	0.98	0.328
Evening-type	0.01	0.06	0.11	0.911
Smooth terms	EDF	RefDF	F-value	*p*-value
ID	345.50	407	5.82	<2e-16***
Time-of-day	3.76	4	29.01	<2e-16***
SD construct	1.00	4	2460.30	<2e-16***
SD construct x time-of-day	0.00	16	0.00	0.836
Time-of-day x morning-type	0.47	4	0.23	0.168
Time-of-day x intermediate-type	0.00	4	0.00	0.919
Time-of-day x evening-type	0.53	4	0.32	0.143
SD construct x morning-type	0.00	4	0.00	0.960
SD construct x intermediate-type	0.00	4	0.00	0.943
SD construct x evening-type	0.00	4	0.00	0.911
SD construct x time-of-day x morning-type	2.10	16	0.66	0.007**
SD construct x time-of-day x intermediate-type	0.00	16	0.00	0.691
SD construct x time-of-day x evening-type	2.44	16	0.96	0.007**

Note. EDF = effective degrees of freedom; RefDF = reference degrees of freedom. Morning-type served as reference category for the parametric coefficients.

Table S44. GAMM results of the associations of fatigue-related symptoms with Social Dysfunction (SD) construct, chronotype, and time-of-day.

Social dysfunction (SD) construct – Fatigue-related symptoms				
Parametric coefficients	Estimate	SE	T value	*p*-value
Intercept	3.28	0.14	23.02	<2e-16***
Intermediate-type	0.16	0.16	1.00	0.317
Evening-type	-0.03	0.19	-0.14	0.889
Smooth terms	EDF	RefDF	F-value	*p*-value
ID	306.50	407	3.13	<2e-16***
Time-of-day	3.78	4	149.29	<2e-16***
SD construct	0.95	4	65.80	0.000***
SD construct x time-of-day	1.84	16	0.23	0.067
Time-of-day x morning-type	1.88	4	31.23	<2e-16***
Time-of-day x intermediate-type	0.99	4	19.63	<2e-16***
Time-of-day x evening-type	0.00	4	0.00	0.396
SD construct x morning-type	0.00	4	0.00	0.817
SD construct x intermediate-type	1.49	4	20.43	0.020*
SD construct x evening-type	0.00	4	0.00	0.992
SD construct x time-of-day x morning-type	2.48	14	1.92	0.000***
SD construct x time-of-day x intermediate-type	1.24	16	0.28	0.007**
SD construct x time-of-day x evening-type	1.34	14	0.41	0.023*

Note. EDF = effective degrees of freedom; RefDF = reference degrees of freedom. Morning-type served as reference category for the parametric coefficients.

Table S45. GAMM results of the associations of low drive and motivation with Social Dysfunction (SD) construct, chronotype, and time-of-day.

Social Dysfunction (SD) construct – Low drive and motivation				
Parametric coefficients	Estimate	SE	T value	*p*-value
Intercept	3.45	0.11	30.80	<2e-16***
Intermediate-type	-0.20	0.13	-1.59	0.112
evening-type	-0.61	0.15	-4.05	0.000***
Smooth terms	EDF	RefDF	F-value	*p*-value
ID	295.90	407	2.83	<2e-16***
Time-of-day	2.74	4	46.13	<2e-16***
SD construct	0.98	4	140.52	0.002**
SD construct x time-of-day	2.25	16	0.28	0.106
Time-of-day x morning-type	0.98	4	12.74	<2e-16***
Time-of-day x intermediate-type	1.57	4	3.74	0.000****
Time-of-day x evening-type	0.00	4	0.00	0.467
SD construct x morning-type	0.00	4	0.00	0.943
SD construct x intermediate-type	0.00	4	0.00	0.899
SD construct x evening-type	0.00	4	0.00	0.957

Note. EDF = effective degrees of freedom; RefDF = reference degrees of freedom. Morning-type served as reference category for the parametric coefficients.

Table S46. GAMM results of the associations of ADHD-type symptoms with Social Dysfunction (SD) construct, chronotype, and time-of-day.

Social dysfunction (SD) construct – ADHD-type symptoms				
Parametric coefficients	Estimate	SE	T value	*p*-value
Intercept	0.68	0.04	17.35	<2e-16***
Intermediate-type	0.04	0.04	0.97	0.334
Evening-type	-0.02	0.05	-0.36	0.722
Smooth terms	EDF	RefDF	F-value	*p*-value
ID	345.80	407	5.87	<2e-16***
Time-of-day	2.50	4	7.80	0.000***
SD construct	1.00	4	1892.06	<2e-16***
SD construct x time-of-day	0.00	16	0.00	0.550
Time-of-day x morning-type	0.49	4	0.26	0.157
Time-of-day x intermediate-type	0.00	4	0.00	0.995
Time-of-day x evening-type	0.97	4	1.18	0.029*
SD construct x morning-type	0.00	4	0.00	0.400
SD construct x intermediate-type	0.00	4	0.00	0.985
SD construct x evening-type	0.39	4	2.70	0.378
SD construct x time-of-day x morning-type	0.73	16	0.11	0.138
SD construct x time-of-day x intermediate-type	0.91	16	0.10	0.149
SD construct x time-of-day x evening-type	0.90	14	0.86	0.002**

Note. EDF = effective degrees of freedom; RefDF = reference degrees of freedom. Morning-type served as reference category for the parametric coefficients.

SI References

1 Oppenheimer DM, Meyvis T, Davidenko N. Instructional manipulation checks: Detecting satisficing to increase statistical power. *J Exp Soc Psychol* 2009. doi:10.1016/j.jesp.2009.03.009.

2 Roenneberg T, Wirz-Justice A, Merrow M. Life between clocks: Daily temporal patterns of human chronotypes. *J Biol Rhythms* 2003; **18**: 80–90.

3 Natale V, Esposito MJ, Martoni M, Fabbri M. Validity of the reduced version of the Morningness-Eveningness Questionnaire. *Sleep Biol Rhythms* 2006; **4**: 72–74.

4 Cole JC, Rabin AS, Smith TL, Kaufman AS. Development and validation of a Rasch-derived CES-D short form. *Psychol Assess* 2004; **16**: 360–372.

5 Löwe B, Decker O, Müller S, Brähler E, Schellberg D, Herzog W *et al.* Validation and standardization of the generalized anxiety disorder screener (GAD-7) in the general population. *Med Care* 2008; **46**: 266–74.

6 Altman EG, Hedeker D, Peterson JL, Davis JM. The altman self-rating Mania scale. *Biol Psychiatry* 1997; **42**: 948–955.

7 Peters E, Joseph S, Day S, Garety P. Measuring delusional ideation: The 21-item Peters et al. Delusions Inventory (PDI). *Schizophr Bull* 2004; **30**: 1005–22.

8 Mason O, Claridge G. The Oxford-Liverpool Inventory of Feelings and Experiences (O-LIFE): Further description and extended norms. *Schizophr Res* 2006; **82**: 203–211.

9 Bjureberg J, Ljótsson B, Tull MT, Hedman E, Sahlin H, Lundh LG *et al.* Development and Validation of a Brief Version of the Difficulties in Emotion Regulation Scale: The DERS-16. *J Psychopathol Behav Assess* 2016; **38**: 284–296.

10 Booth T, Murray AL, McKenzie K, Kuenssberg R, O'Donnell M, Burnett H. Brief report: An evaluation of the AQ-10 as a brief screening instrument for asd in adults. *J Autism Dev Disord* 2013; **43**: 2997–3000.

11 Gustavsson JP, Jönsson EG, Linder J, Weinryb RM. The HP5 inventory: Definition and assessment of five health-relevant personality traits from a five-factor model perspective. *Personal Individ Differ* 2003; **35**: 69–89.

12 Oliver MNI, Simons JS. The affective lability scales: Development of a short-form measure. *Personal Individ Differ* 2004; **37**: 1279–1288.

13 Adler LA, Spencer T, Faraone SV, Kessler RC, Howes MJ, Biederman J *et al.* Validity of pilot adult ADHD Self-Report Scale (ASRS) to rate adult ADHD symptoms. *Ann Clin Psychiatry* 2006; **18**: 145–8.

14 Foa EB, Huppert JD, Leiberg S, Langner R, Kichic R, Hajcak G *et al.* The Obsessive-Complusive Inventory: Development and validation of a short version. *Psychol Assess* 2002; **14**: 485–96.

15 Garner DM, Bohr Y, Garfinkel PE. The Eating Attitudes Test: Psychometric Features and Clinical Correlates. *Psychol Med* 1982; **12**: 871–878.

16 Marin RS, Biedrzycki RC, Firinciogullari S. Reliability and validity of the apathy evaluation scale. *Psychiatry Res* 1991; **38**: 143–62.

17 Heimberg RG, Horner KJ, Juster HR, Safren SA, Brown EJ, Schneier FR *et al.* Psychometric properties of the Liebowitz Social Anxiety Scale. *Psychol Med* 1999; **29**: 199–212.

18 Liebowitz MR. Liebowitz Social Anxiety Scale. *Mod Probl Pharmapsychiatry* 1987.

19 Gillan CM, Kosinski M, Whelan R, Phelps EA, Daw ND. Characterizing a psychiatric symptom dimension related to deficits in goal directed control. *eLife* 2016; **5**: e11305.

20 Dalgleish T, Black M, Johnston D, Bevan A. Transdiagnostic approaches to mental health problems: Current status and future directions. *J Consult Clin Psychol* 2020; **88**: 179–195.

21 Vindegaard N, Benros ME. COVID-19 pandemic and mental health consequences: Systematic review of the current evidence. *Brain Behav Immun* 2020; **89**: 531–542.

22 Rouault M, Seow T, Gillan CM, Fleming SM. Psychiatric Symptom Dimensions Are Associated With Dissociable Shifts in Metacognition but Not Task Performance. *Biol Psychiatry* 2018. doi:10.1016/j.biopsych.2017.12.017.
